# Oral Exposure to Food‐Grade Nanoparticles Poses a Risk of Alzheimer's Disease‐Like Symptoms by Triggering Autophagy Defects in Neurons

**DOI:** 10.1002/advs.202508096

**Published:** 2025-10-27

**Authors:** Jiaxin Shang, Jun Yan, He Lou, Xuanxi Jiang, Ziyue Wang, Yue Gao, Xiaohui Fan, Xiaoyan Lu

**Affiliations:** ^1^ Pharmaceutical Informatics Institute, College of Pharmaceutical Sciences Zhejiang University Hangzhou 310058 China; ^2^ State Key Laboratory of Chinese Medicine Modernization Innovation Center of Yangtze River Delta Zhejiang University Jiaxing 314102 China; ^3^ Jinhua Institute of Zhejiang University Jinhua 321299 China; ^4^ Department of Pharmaceutical Sciences Beijing Institute of Radiation Medicine Beijing 100850 China

**Keywords:** Alzheimer's disease, autophagy defects, DNA methylation, food‐grade nanoparticles, potential neurotoxicity

## Abstract

Preliminary epidemiological studies have revealed a relationship between exposure to environment‐related ultrafine particles and the escalation of Alzheimer's disease (AD). Oral exposure through food is a significant route of human contact with nanoparticles; however, the potential risk of AD induced by food‐grade nanoparticles and the underlying mechanisms remain largely unclear. Here, this study reveals a common mechanism by which food‐grade nanoparticles, including titanium dioxide, nanosilica, and nanosilver, trigger AD‐like pathological changes through epigenetic alterations. Exposure to food‐grade nanoparticles triggers changes in DNA methylation and aberrant ryanodine receptor‐Ca^2+^ signaling in the mouse brain, contributing to lysosomal impairment and disrupted autophagic flux in neurons. Crucially, these autophagy defects reduced the ability to clear β‐amyloid and pTau proteins, which ultimately accumulated and triggered spatial cognition and memory deficits in mice. In conclusion, this study elucidates the shared toxicological mechanisms induced by different food‐grade nanoparticles, thereby offering valuable insights into ingested nanoparticle exposure and its potential association with neurodegenerative diseases.

## Graphical abstract

Three food‐grade nanomaterials induce dysregulated RyR‐Ca^2+^ signaling through epigenetic modifications. This dysregulation impairs the autophagic clearance process, ultimately leading to abnormal Aβ deposition and increase phosphorylated tau levels. These common toxic mechanisms highlight a critical link between nanoparticle exposure and neurodegenerative diseases, further driving attention to the potential risk of food‐grade nanoparticles in contributing to Alzheimer's disease.

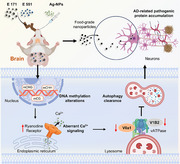

## 1 Introduction

Recent epidemiological data have shown that exposure to traffic‐related air pollution (TRAP; mainly ultrafine particles) is associated with an increased risk of Alzheimer's disease (AD) and promotes AD phenotypes in wild‐type rats.^[^ ^1^, ^2^ ^]^ Nanoscale particles have been detected in the hippocampus of TRAP‐exposed animals, which suggests that these particles may be an important factor in increasing AD risk.^[^ ^1^ ^]^ Moreover, a greater accumulation of nanoplastics has been observed in the decedent brains with AD, thereby further revealing the potential association between nanoparticles and the onset and escalation of AD.^[^ ^3^ ^]^ Beyond these, nanoparticles in food pose a potentially greater health risk due to unintentional oral exposure.^[^ ^4^ ^]^ However, the potential risks and underlying mechanisms of AD associated with human exposure to nanoparticles via oral intake remain poorly understood.

Growing evidence highlights environmental factors that may trigger or exacerbate the onset of AD.^[^ ^5^, ^6^, ^7^ ^]^ Environmental exposure is often associated with disease pathogenesis via epigenetic changes, particularly DNA methylation alterations.^[^ ^8^ ^]^ DNA methylation changes are considered a sensitive indicator for detecting toxicity induced by nanoparticle exposure.^[^ ^9^ ^]^ This suggests that changes in DNA methylation have significant potential to reveal how environmental influences, including nanoparticle exposure, contribute to AD development.

Abnormal Ca^2+^ signaling is regarded as a central pathological event in AD development.^[^ ^10^ ^]^ Multiple Ca^2+^ channels and signal transduction systems are altered in AD, including excessive Ca^2+^ release from the endoplasmic reticulum mediated by the ryanodine receptor (RyR).^[^ ^11^ ^]^ Notably, an abnormal increase in RyR‐Ca^2+^ signaling leads to the defect of autophagy and overaccumulation of phosphorylated tau (pTau) protein.^[^ ^12^ ^]^ This demonstrates that normal RyR‐Ca^2+^ signaling and autophagy are crucial in eliminating AD‐related pathogenic proteins. Moreover, numerous nanoparticles have been shown to regulate the autophagic process, including mediating autophagy defects.^[^ ^13^ ^]^ Therefore, we hypothesized that autophagy defects triggered by nanoparticle exposure might contribute to the accumulation of AD‐related pathogenic proteins.

**Scheme 1:**
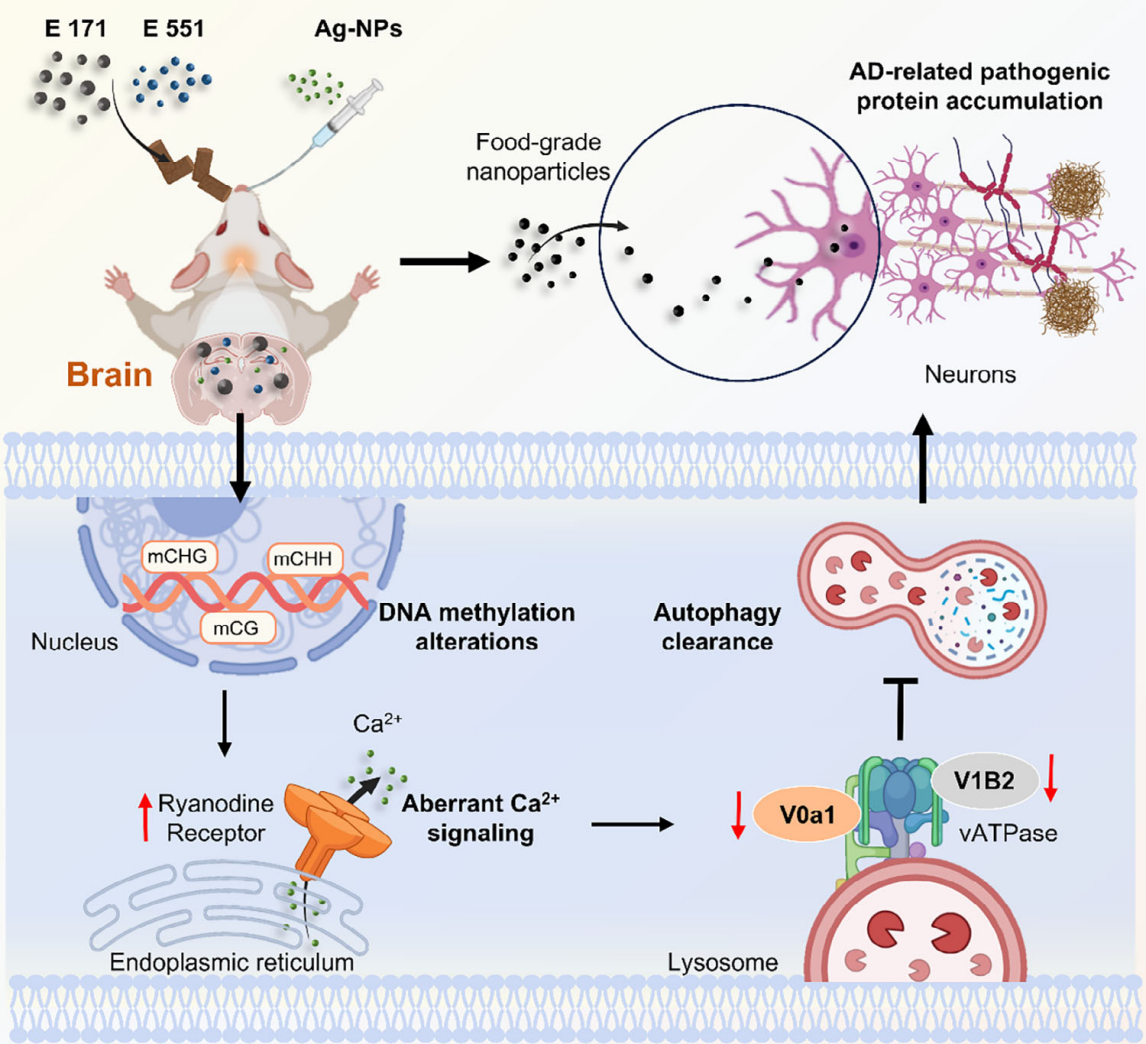
Schematic illustration of three food‐grade nanoparticles inducing autophagy defects via epigenetic modifications, leading to the accumulation of AD‐associated pathogenic proteins and the development of AD‐like symptoms. Created with BioRender.com.

Herein, we report that subchronic (84‐day) exposure to food‐grade nanoparticles induces AD‐like pathological changes in mice. Given the widespread use and exposure potential of food‐ and pharmaceutical‐grade TiO_2_ (E 171) as a colorant and film‐coating agent,^[^ ^14^, ^15^ ^]^ we first examined the epigenetic changes triggered by two types of E 171 in the mouse brain by performing a whole‐genome bisulfite sequencing (WGBS) analysis. Food‐grade nanosilica (E 551) and dietary supplement nanosilver (Ag‐NPs), which serve as anti‐caking agents and antimicrobials, respectively, were used to investigate the common toxicity mechanisms of food‐grade nanoparticles.^[^ ^16^, ^17^ ^]^ Exposure to E 171 triggered pathological and epigenetic changes in the mouse brain, and WGBS revealed the risk of AD instigated by E 171 via RyR‐Ca^2+^ signaling. E 171 entering the mouse brain induced autophagy defects, cognitive and memory deficits, and AD‐like pathological changes via abnormally increased RyR‐Ca^2+^ signaling. Notably, E 551 and Ag‐NPs produced common toxic mechanisms and effects as E 171, both in vitro and in vivo (Scheme 1). These findings highlight the importance of assessing and preventing risks associated with nanoparticle exposure, especially via oral exposure.

## 2 Results and Discussion

### 2.1 Subchronic Exposure to E 171 Induces Pathological and DNA Methylation Alterations in Mouse Brain

**Figure 1:**
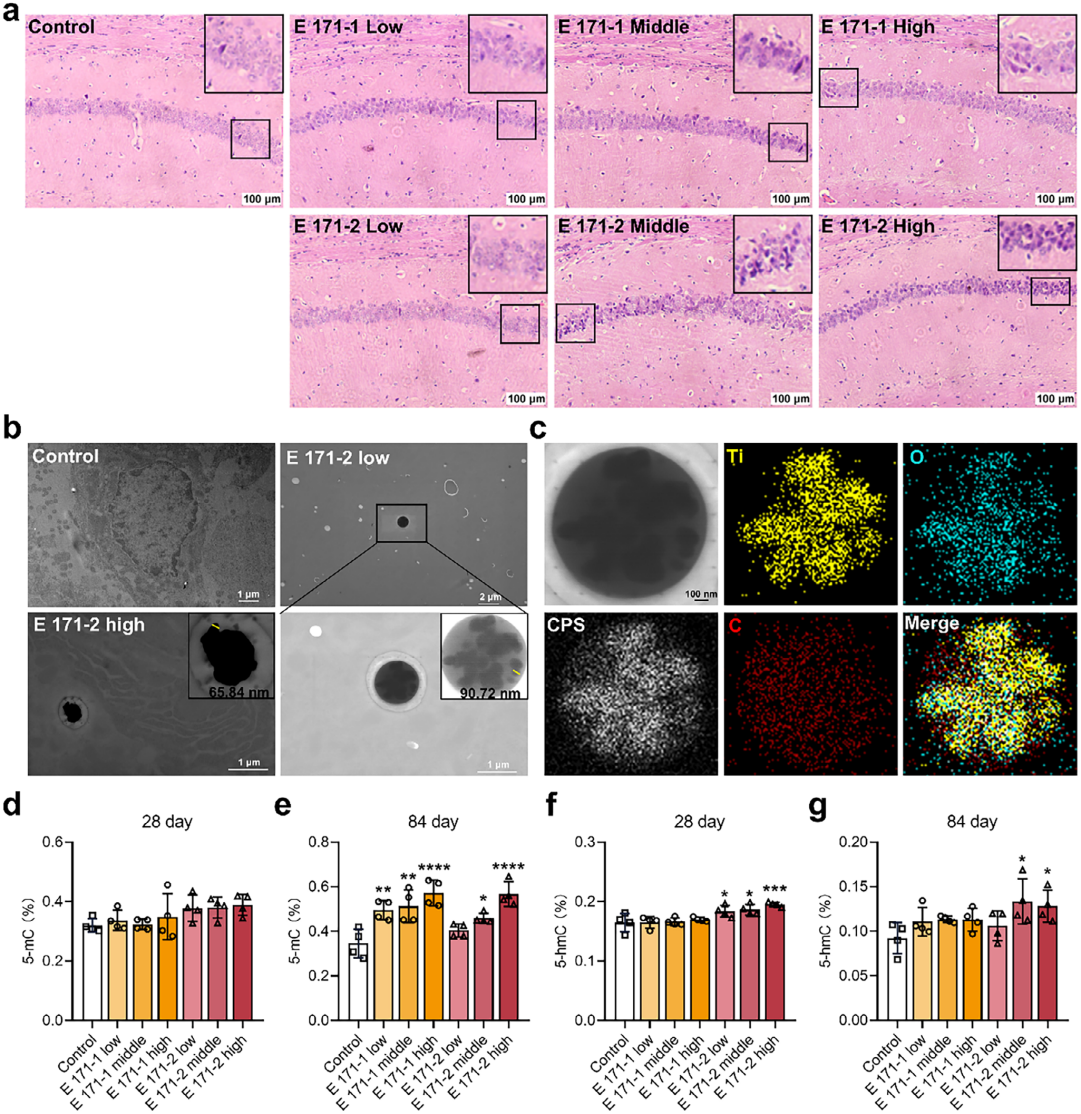
E 171 entering the brain triggers pathological and epigenetic changes. a) H&E analysis of the brain of mice exposed to E 171‐1 and E 171‐2 for 84 days. *n* = 4; *n* indicates the number of mice in each group. b) Representative images of hippocampal regions in mice after 84 days of E 171 exposure, obtained by SEM. c) EDS‐based elemental analysis: yellow for Ti, blue for O, and red for C. d) Global 5‐mC levels in the brain of mice exposed to E 171 for 28 days. e) Global 5‐mC levels in the brain of mice exposed to E 171 for 84 days. f) Global 5‐hmC levels in the brain of mice exposed to E 171 for 28 days. g) Global 5‐hmC levels in the brain of mice exposed to E 171 for 84 days. Statistical analysis was performed through one‐way ANOVA followed by Dunnett's test. Compared with the control group, ^*^ *p* < 0.05, ^**^ *p* < 0.01, ^***^ *p* < 0.001, ^****^ *p* < 0.0001.

Two types of E 171 (E 171‐1 and E 171‐2) were selected to assess potential neurotoxicity, with their characterizations detailed in our previous study.^[^ ^18^ ^]^ Both E 171‐1 and E 171‐2 exhibit the anatase crystal structure commonly used in food and pharmaceutical applications, with average minimum Feret diameters of 111.8 and 123.9 nm, respectively. E 171‐1 is widely employed in various foods, while E 171‐2 complies with international regulatory standards for use in both food and pharmaceutical applications. E 171‐2 is extensively applied in food products and pharmaceutical formulations, particularly in oral solid dosage forms. Mice were orally exposed to E 171‐1 and E 171‐2 for 28 days (subacute exposure) and 84 days (subchronic exposure), respectively, to assess phenotypic changes in mouse brains (Figure S1a, Supporting Information). The doses for the low‐ and middle‐dose (8, 80 mg kg^−1^ bw per day) groups were determined from the estimated adult intake of E 171 (0.6−6.8 mg kg^−1^ bw per day, where 0.6 and 6.8 correspond to 8 and 80 mg kg^−1^ bw per day, respectively) as assessed by the European Food Safety Authority (EFSA), after normalization to the body surface area of human and mouse.^[^ ^14^, ^19^ ^]^ High‐dose groups (320 mg kg^−1^ bw per day) were designed to assess a broader range of exposure scenarios in addition to those involving food. Hematoxylin and eosin (H&E) staining of mouse brains after exposure to E 171‐1 and E 171‐2 for 84 days revealed nuclear pyknosis and karyolysis in hippocampal CA1 cells, which is a crucial region for learning and memory.^[^ ^20^, ^21^ ^]^ These changes were observed after 84 days of exposure to middle‐ and high‐dose E 171‐1 and E 171‐2 (**Figure** **1**a). To determine whether oral administration of E 171 leads to accumulation in the mouse brain, we first examined the cortical region following 84 days of exposure to E 171‐1 and E 171‐2 using scanning electron microscopy (SEM) and energy‐dispersive X‐ray spectroscopy (EDS). The results revealed the presence of individual TiO_2_ particles or TiO_2_ aggregates in the cortical region across all exposure groups (Figure S1c, d, Supporting Information). We further examined the hippocampal region after low‐ and high‐dose E 171‐2 exposure and found an accumulation of TiO_2_ particles (Figure 1b; Figure S1e, Supporting Information). Notably, we found dozens of TiO_2_ particles encapsulated in membrane structures in the hippocampal region (Figure 1b,c). This suggests that TiO_2_ particles in E 171 are transported to the brain in large quantities through oral exposure, even at levels below the average human exposure dose (1 mg kg^−1^ bw per day of Ti).^[^ ^22^ ^]^ Studies have demonstrated that E 171 is absorbed through the intestinal tract following oral exposure, enters the bloodstream, and subsequently reaches vital organs such as the liver and lungs.^[^ ^23^, ^24^, ^25^ ^]^ Absorption of E 171 from the jejunal and ileal villi peaks at 4 h after E 171 exposure (40 mg kg^−1^ bw), with a delayed uptake observed in the jejunal Peyer's patches at 8 h.^[^ ^23^ ^]^ Additionally, E 171 translocation occurs primarily via passive paracellular diffusion and a goblet cell‐associated pathway.^[^ ^23^ ^]^ A study in rats confirmed that E 171 particles (10 mg kg^−1^ bw for 7 days) accumulate in Peyer's patches, colonic mucosa, and liver following repeated oral exposure.^[^ ^24^ ^]^ In this study, E 171 was also detected in the cerebral cortex and hippocampus after subchronic exposure, indicating a broader biodistribution and potential neurotoxic effects.

Epigenetic modifications, especially DNA methylation, are closely associated with disease development,^[^ ^26^ ^]^ and our previous studies revealed that E 171 and E 551 can induce epigenetic toxicity in the liver.^[^ ^16^, ^18^ ^]^ To assess the epigenetic changes induced by TiO_2_ particles entering the brain, we examined the expression levels of 5‐methylcytosine (5‐mC), 5‐hydroxymethylcytosine (5‐hmC), methylation elements, and transposable factors in the brains of mice after exposure to E 171 for 28 and 84 days. After 28 days of E 171‐1 or E 171‐2 exposure, 5‐mC levels in the brain showed no significant difference compared to controls (Figure 1d). However, 5‐mC levels were significantly increased in the brains of mice treated with E 171‐1 and E 171‐2 for 84 days compared with those in the control group (Figure 1e). Additionally, 5‐hmC levels in the brain were significantly increased in a dose‐dependent manner after subacute exposure to E 171‐2 (Figure 1f). A similar increase in 5‐hmC levels was observed after E 171‐2 exposure for 84 days (Figure 1g). Elevated levels of 5‐mC and 5‐hmC in the brain may be due to increased gene expression of the *Dnmt* (*Dnmt1* and *Dnmt3a*) and *Tet* (*Tet1*, *Tet2*, and *Tet3*) families (Figure S2a, Supporting Information). Additionally, the expression levels of several transposable elements, including *LINE‐1 ORF1*, *SINEB1*, and *SINEB2*, were significantly down‐regulated in the brain after middle‐ and high‐dose E 171‐2 exposure (Figure S2b, Supporting Information).

In conclusion, our study demonstrates that subacute and subchronic exposure to E 171‐1 and E 171‐2 leads to TiO_2_ accumulation, histopathological alterations, and epigenetic modifications in mouse brains. Notably, existing studies reveal additional toxicological profiles for E 171‐1 and E 171‐2. Zhu et al. demonstrated that long‐term oral exposure to E 171‐1 exacerbated atherosclerosis in APOE^−/−^ mice, potentially through gut microbiota‐mediated choline metabolism disruption.^[^ ^27^ ^]^ Jayaram et al. reported that E 171‐2 triggered intracellular superoxide production and altered HDAC9/10 expression in lung cells, indicating oxidative stress and epigenetic modulation.^[^ ^28^ ^]^ Together with our findings, these studies suggest that both forms of E 171 may exert broader systemic toxic effects and warrant a more comprehensive safety evaluation.

### 2.2 E 171 Induces DNA Methylation Landscape Changes in the Brain and Increases the Risk of Neurodegenerative Diseases

**Figure 2:**
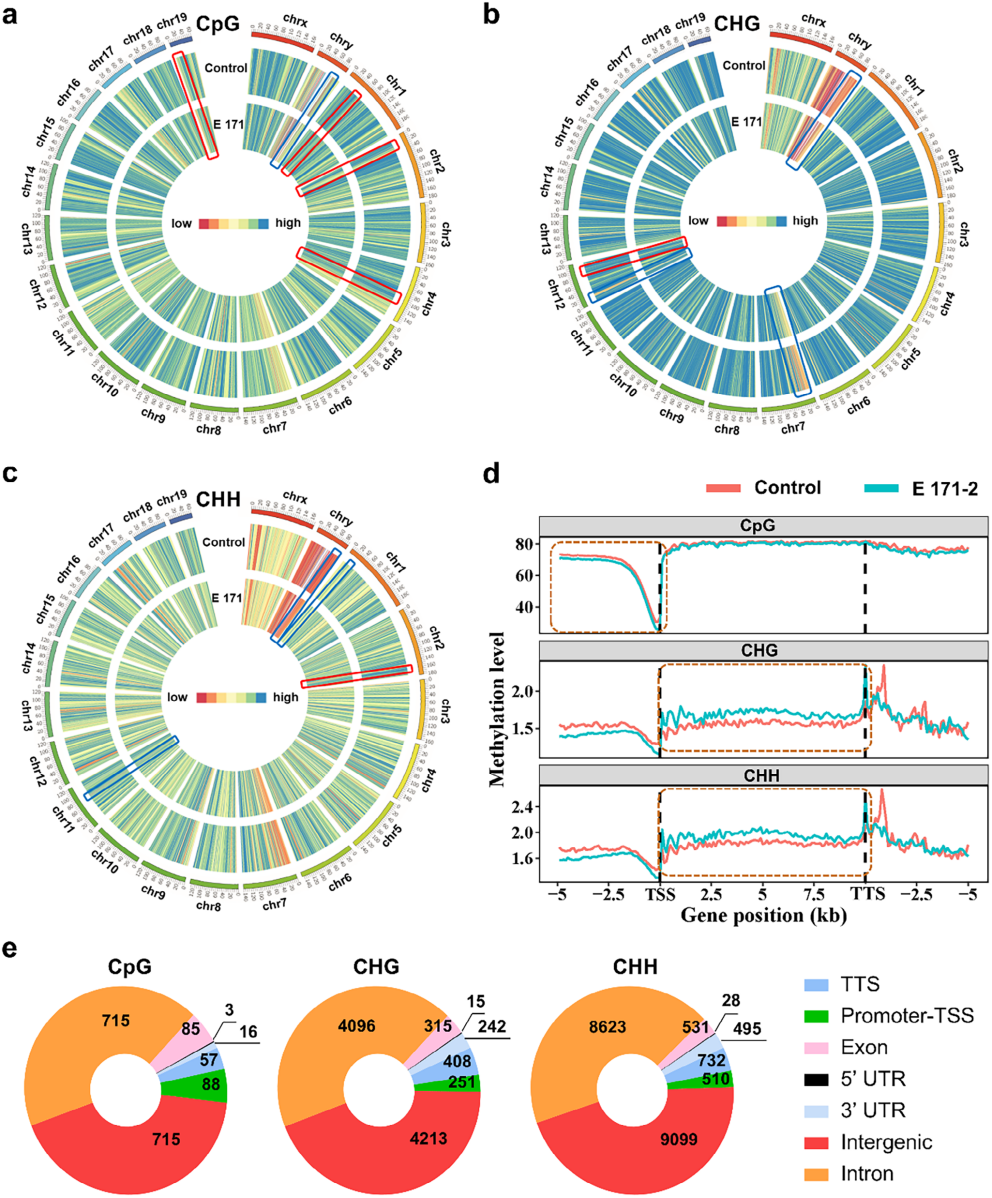
Genomic DNA methylation landscape in the brain. a–c) Circos plots of mCpG (a), mCHG (b), and mCHH (c) methylation levels on 21 chromosomes of the whole genome. Hypermethylation regions are shown in the blue boxes, and hypomethylation regions are shown in the red boxes. d) Distribution of DNA methylation changes in different gene regions (TSS, gene body, TES, and 5‐kb upstream and downstream). e) Distribution and proportion of DMRs in the mCpG, mCHG, and mCHH contexts.

We selected the middle‐dose E 171‐2 group as the representative exposure group for subsequent WGBS analyses for the following reasons: 1) both middle‐ and high‐dose E 171‐2 exposures induced significant pathological and epigenetic alterations in the mouse brain; 2) based on the conversion of body surface area,^[^ ^19^ ^]^ the dosage of the middle‐dose E 171‐2 group (80 mg kg^−1^ bw per day) fell within the range of potential human exposure levels (0.6−6.8 mg kg^−1^ bw per day) and was more representative of the exposure scenarios likely to be encountered by humans.^[^ ^14^ ^]^ The quality of raw sequencing data and clean reads after WGBS indicated that these samples satisfied the quality threshold for further analyses (Table S1, Supporting Information). Principal component analysis of methylation levels of CpG sites in each sample revealed obvious DNA methylation differences in samples between the control and middle‐dose E 171‐2 groups (Figure S2c, Supporting Information). Circos plots displayed DNA methylation levels in the mCG, mCHG, and mCHH contexts across brain chromosomes. Several alterations were observed in the mCG context, including hypermethylation at 65–70 Mb on chromosome Y, and hypomethylation at 40–45 Mb on chromosome 1, 20–25 Mb on chromosome 2, 90–100 Mb on chromosome 4, and 5–10 Mb on chromosome 19 (**Figure** **2**a). Hypermethylation and hypomethylation were also detected in several regions within the mCHG and mCHH contexts, thereby suggesting that E 171 exposure significantly affects non‐CG sites in the brain and warrants further attention (Figure 2b,c). Additionally, a slight decrease in mCG transcription start sites (TSS) was observed in the brains of mice exposed to middle‐dose E 171‐2 for 84 days. Methylation levels in the gene body and transcription termination sites (TTS) regions were considerably higher in the mCHG and mCHH contexts after E 171‐2 exposure (Figure 2d).

Differentially methylated sites (DMSs), regions (DMRs), and genes (DMGs) were identified as detailed in our previous study.^[^ ^18^ ^]^ A heatmap of DMSs in the mCG context showed that subchronic exposure to E 171 caused considerable methylation alterations in the mouse brain (Figure S2d, Supporting Information). Furthermore, 1679 DMRs were identified in the mCG context, primarily in intron and intergenic regions, followed by promoter regions. In the mCHG and mCHH contexts, 9540 and 20018 DMRs were detected, respectively, and these were primarily located within intergenic and intron regions (Figure 2e). Notably, the number of DMRs detected in the mCHG and mCHH contexts was higher than in the mCpG context, which included promoter regions that regulate gene expression.^[^ ^29^ ^]^ Specifically, 88, 251, and 510 DMRs were identified in promoter regions within the mCpG, mCHG, and mCHH contexts, respectively (Figure 2e). These findings suggest that subchronic exposure to E 171 induces DNA methylation alterations in the mouse brain across both CG and non‐CG contexts, with more pronounced methylation changes occurring in the non‐CG context. Notably, previous studies, including our own, have confirmed that E 171 exposure induces global DNA methylation changes in mouse colon epithelial cells and the liver.^[^ ^18^, ^30^ ^]^ Combined with the findings of the present study, this suggests that E 171 may exert broad regulatory effects on DNA methylation. Furthermore, our research and other studies have demonstrated that exposure to nanomaterials such as E 551 and graphene oxide also triggers alterations in the methylation levels of the non‐CG context.^[^ ^16^, ^31^ ^]^ These findings indicate that food‐grade nanomaterials may possess extensive epigenotoxic potential by modulating methylation patterns in both CG and non‐CG contexts, thereby highlighting the urgent need for further in‐depth investigations into epigenotoxic mechanisms.

The biological functions of DMGs in the promoter regions within the mCG context were investigated using the Gene Ontology (GO) and Kyoto Encyclopedia of Genes and Genomes (KEGG) databases. The cell fate specification pathway was significantly enriched as the top pathway (Figure S3a, Supporting Information). Importantly, the neurodegenerative diseases pathway was significantly enriched (*p* < 0.05) after subchronic exposure to E 171 (Figure S3b, Supporting Information). Notably, nanoparticles have been proven to be closely associated with the onset and progression of various neurodegenerative diseases, including AD, Parkinson's disease (PD), and amyotrophic lateral sclerosis (ALS).^[^ ^32^, ^33^, ^34^ ^]^ In addition, pathway enrichment analysis of DMGs in promoter regions within the mCHG and mCHH contexts revealed significant enrichment in pathways such as IL‐17 signaling, regulation of calcium ion transport, regulation of intracellular pH, and negative regulation of autophagy (Figure S3c,d, Supporting Information). These pathways are closely linked to neurodegenerative diseases, especially AD.^[^ ^10^, ^12^, ^35^ ^]^ These findings suggest that E 171‐induced epigenetic changes in mouse brains may heighten the risk of neurodegenerative diseases, with dysregulated Ca^2^⁺ signaling and autophagy playing key mechanistic roles.

### 2.3 E 171 Carries the Risk of AD‐Like Symptoms

**Figure 3:**
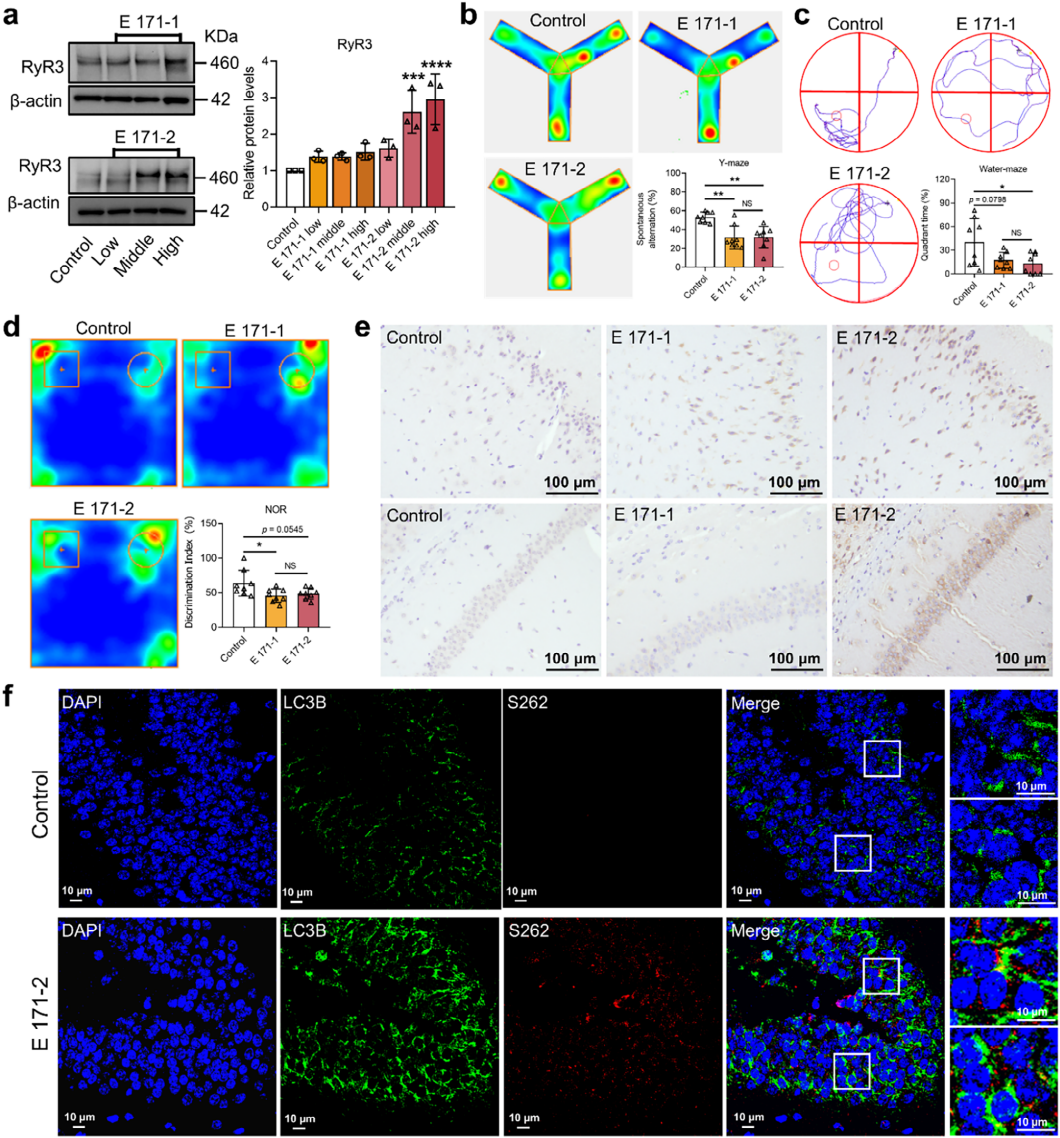
Subchronic exposure to E 171 causes spatial recognition and memory deficits, as well as AD‐like pathology in mice, through RyR‐mediated autophagy defect. a) Changes in protein expression of RyR3 in the brain. *n* = 4; *n* indicates the number of mice in each group. Statistical analysis was performed through one‐way ANOVA followed by Dunnett's test. b) Thermal map and spontaneous alternation rate of mice in the Y‐maze after E 171 exposure compared with those in the control group. *n* = 8; *n* indicates the number of mice in each group. Statistical analysis was performed through one‐way ANOVA with Tukey's multiple comparison tests. c) Representative mouse roadmap and residence time of mice in the platform quadrant in the water maze experiment. *n* = 8; *n* indicates the number of mice in each group. Statistical analysis was performed through one‐way ANOVA with Tukey's multiple comparison tests. d) Thermal map and discrimination index of mice in NOR after E 171 exposure compared with those in the control group. *n* = 8; *n* indicates the number of mice in each group. Circles (○) indicate the familiar object (cylindrical rod) presented during the training phase, while squares (□) represent the novel object (rectangular block) introduced during the testing phase. Statistical analysis was performed through one‐way ANOVA with Tukey's multiple comparison tests. e) Representative immunohistochemistry images of Aβ 1–42 in the brain of mice. f) Representative immunofluorescence images of LC3B and pTau(S262) in the dentate gyrus region of the brain from the control and middle‐dose E 171‐2 groups. Compared with the control group, ^*^ *p* < 0.05, ^**^ *p* < 0.01, ^***^ *p* < 0.001, ^****^ *p* < 0.0001.

The DMGs affected in the “neurodegenerative disease” and “cell fate specification” pathways were further analyzed. In the “neurodegenerative disease” pathway, the promoter region of ryanodine receptor 3 (*RyR3*) was hypomethylated. Bisulfite‐pyrosequencing methylation analysis of the *RyR3* revealed that 10 out of 13 examined CpG sites in the promoter region showed reduced methylation levels following middle‐dose E 171‐2 exposure for 84 days (Figure S3e, Supporting Information). Increased RyR expression‐mediated Ca^2+^ release affects the clearance of AD‐related pathogenic proteins in mouse neurons.^[^ ^12^ ^]^ Therefore, we speculate that the E 171‐induced reduction in *RyR3* methylation levels may increase RyR3 gene and protein expression, thereby contributing to the accumulation of AD‐related pathogenic proteins in the brain and to cognitive dysfunction. As expected, the gene and protein expression levels of RyR3 were consistently and significantly elevated, especially in the middle‐ and high‐dose E 171‐2 groups (**Figure** **3**a; Figure S3f, Supporting Information). Furthermore, analysis of the “cell fate specification” pathway revealed significant hypomethylation of the *FEV* gene in its promoter region. Notably, *FEV* is a key cascade factor in initiating serotonin synthesis,^[^ ^36^ ^]^ the serotonin may potentiate abnormal neuronal Ca^2+^ signaling.^[^ ^37^ ^]^ The protein expression of FEV was significantly increased after middle‐ and high‐dose E 171‐2 exposure (Figure S3g, Supporting Information).

AD is characterized by memory impairment and cognitive decline.^[^ ^38^ ^]^ To assess the cognition and memory ability of mice exposed to E 171, the Y‐maze, water maze, and novel object recognition (NOR) assays were performed after middle‐dose E 171‐1 or E 171‐2 exposure (Figure S3h, Supporting Information). Subchronic oral exposure to E 171 was found to cause behavioral abnormalities in mice, including reduced spatial recognition and memory abilities, particularly long‐term memory (Figure 3b–d). In addition, no significant differences were observed between groups in the total number of arm entries in the Y‐maze, or in the total distance traveled in the water maze and NOR area. These findings indicate that subchronic exposure to E 171 did not affect the motor abilities of the mice, and that observed impairments in memory and recognition were not attributable to deficits in motor function (Figure S4a–c, Supporting Information). Considering that subchronic exposure to E 171 induced nuclear pyknosis and karyolysis in hippocampal CA1 cells, and that apoptosis is an important pathological feature of AD, additional terminal deoxynucleotidyl transferase‐mediated dNTP nick end labeling (TUNEL) staining was performed on the brains of middle‐dose E 171‐2‐exposed mice.^[^ ^39^ ^]^ The results revealed the presence of TUNEL‐positive cells in both the hippocampal CA1 region and the cerebral cortex following E 171‐2 exposure (Figure S4d,e, Supporting Information). As β‐amyloid (Aβ) accumulation and tau lesion spread are hallmarks of early AD,^[^ ^40^ ^]^ we performed immunohistochemical staining for Aβ 1–42. Middle‐dose E 171 exposure resulted in Aβ 1–42 deposits in mouse brains, which were absent in controls (Figure 3e). Since abnormal RyR‐Ca^2+^ signaling may result in the accumulation of hyperphosphorylated tau proteins due to autophagy defects in neurons,^[^ ^12^ ^]^ we further examined LC3B and pTau proteins (S262 and AT8) in the mouse brain after middle‐dose E 171‐2 exposure. The hippocampus and cortex are crucial for learning and memory, and these two areas are easily damaged and affected in the early stages of AD.^[^ ^41^, ^42^ ^]^ Therefore, we primarily focused on the effects of E 171 exposure on the hippocampus and cortex. Immunofluorescence tests showed a considerable increase in LC3B staining in the CA1, dentate gyrus, and cerebral cortex regions of the mouse brain after E 171 exposure, along with significant pTau(S262) positive staining (Figure 3f; Figure S5a,b, Supporting Information). In addition, pTau(AT8) accumulation was also observed in the CA1, dentate gyrus, and cortex regions of the mouse brain (Figure S5c–e, Supporting Information).

### 2.4 E 171 Increases the Accumulation of AD‐Related Pathogenic Proteins in Neurons by Inhibiting Autophagic Clearance

**Figure 4:**
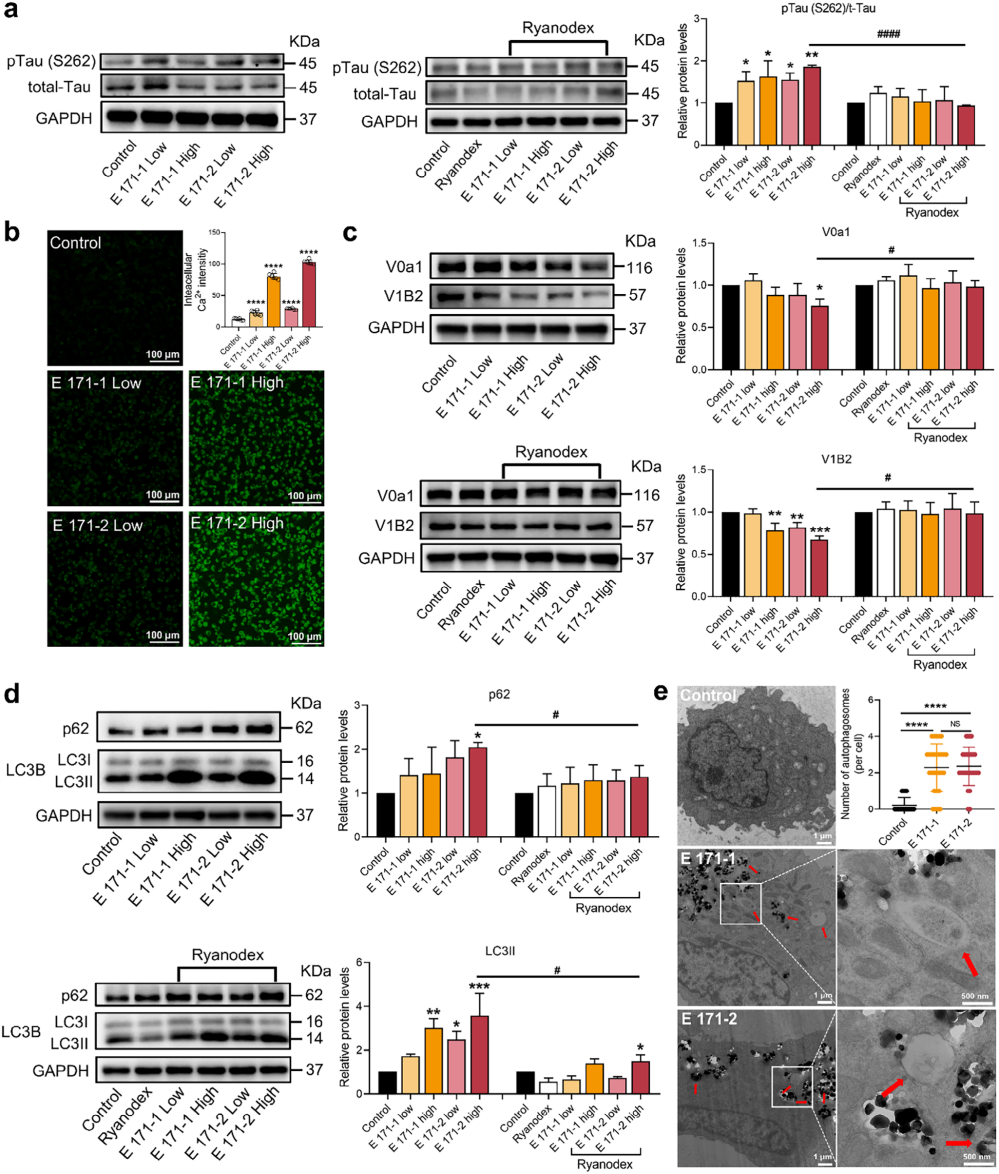
E 171 triggers pTau accumulation in HT22 cells through autophagy defects caused by increased RyR‐Ca^2+^ signaling. a) Changes in protein expression of pTau(S262) and total‐Tau in HT22 cells exposed to E 171‐1 or E 171‐2 (10 and 100 µg mL^−1^) with or without Ryanodex. Statistical analysis was performed through one‐way ANOVA followed by Dunnett's test, and two‐group comparisons were conducted using Student's t‐test (two‐tailed). b) Representative images of Ca^2+^ level in HT22 cells after E 171‐1 or E 171‐2 exposure. Statistical analysis was performed through one‐way ANOVA followed by Dunnett's test. c) Changes in protein expression of V0a1 and V1B2 in HT22 cells exposed to E 171‐1 or E 171‐2 with or without Ryanodex. Statistical analysis was performed through one‐way ANOVA followed by Dunnett's test, and two‐group comparisons were conducted using Student's t‐test (two‐tailed). d) Changes in protein expression of p62 and LC3B in HT22 cells exposed to E 171‐1 or E 171‐2 with or without Ryanodex. Statistical analysis was performed through one‐way ANOVA followed by Dunnett's test, and two‐group comparisons were conducted using Student's t‐test (two‐tailed). e) Representative images of HT22 cells obtained by TEM after exposure to high‐dose E 171‐1 or E 171‐2 (23 cells for the control group and 28 cells for each of the E 171‐exposed groups). Autophagic vesicles are indicated by red arrows. Statistical analysis was performed through two‐tailed Mann–Whitney *U* tests. Compared with the control group, ^*^ *p* < 0.05, ^**^ *p* < 0.01, ^***^ *p* < 0.001, ^****^ *p* < 0.0001. Compared with the E 171‐2 high group, ^#^ *p* < 0.05, ^####^ *p* < 0.0001.

Hippocampus‐derived HT22 cells were exposed to low (10 µg mL^−1^) and high (100 µg mL^−1^) doses of E 171‐1 and E 171‐2 for 24 h to further investigate the mechanism of how E 171 triggers AD‐like pathological changes through RyR‐Ca^2+^ release in neurons. Dose selection was based on HT22 cell viability following 24‐h exposure to E 171‐1 and E 171‐2, with the low and high concentrations corresponding to non‐cytotoxic (≥ 85% cell viability) and sub‐toxic levels (60–85% viability), respectively (Figure S6a, Supporting Information). Food‐grade nanoparticle dispersions for cell exposure were prepared according to the generic Nanogenotox dispersion scheme.^[^ ^43^ ^]^ The hydrodynamic diameter and zeta potentials of both E 171 forms in the complete culture medium are shown in Table S2 (Supporting Information). After E 171‐1 and E 171‐2 exposure, the expression of pTau(S262) was significantly increased in all E 171‐exposed cells, compared to those in the control group (**Figure** **4**a). Treatment with the negative allosteric RyR modulator, Ryanodex (10 µm), restored pTau(S262) expression to normal cellular levels in HT22 cells (Figure 4a), thereby emphasizing the importance of RyR‐Ca^2+^ signaling in the autophagic clearance process. The Fluo‐4 AM assay (used for visualizing and measuring intracellular Ca^2+^) revealed a significant increase in Ca^2+^ in the cellular matrix after E 171 exposure (Figure 4b).

An abnormal increase in RyR‐Ca^2+^ signaling may impair lysosomal acidification by decreasing the expression of lysosomal vacuolar [H^+^] ATPase (vATPase) subunits.^[^ ^12^ ^]^ Therefore, the levels of vATPase subunits (V0a1 and V1B2) were examined and found to be significantly decreased after high‐dose E 171 exposure, while treatment with Ryanodex (10 µm) restored the expression of V0a1 and V1B2 (Figure 4c). Abnormally reduced expression of vATPase subunits is indicative of lysosomal dysfunction. LysoSensor Green and LysoTracker Red were employed to further evaluate the effects of E 171 exposure on lysosomal function and acidification. The results demonstrated that both the fluorescence intensity of LysoSensor Green and the number of LysoTracker Red puncta in HT22 cells were significantly reduced in a dose‐dependent manner following exposure to E 171‐1 and E 171‐2, indicating a decrease in lysosomal abundance and impaired acidification (Figure S6c,d, Supporting Information). Lysosomal dysfunction and insufficient acidification may further impair the normal autophagic process. Following E 171 exposure, both the autophagy substrate p62 and the autophagy marker LC3B were significantly elevated. Additionally, this elevation could be reduced by treatment with Ryanodex (10 µm), suggesting that aberrant RyR‐Ca^2+^ release caused defective autophagic clearance (Figure 4d). Examination of the number of autophagic vesicles after E 171 exposure using transmission electron microscopy (TEM) revealed a significant increase in autophagic vesicle structures (Figure 4e, red arrows). Further examination of autophagic flux, performed by transfecting HT22 cells with Ad‐mCherry‐GFP‐LC3B, revealed that E 171 exposure significantly increased the number of autophagosomes and markedly decreased the number of autolysosomes in neurons. This further confirmed that E 171 induced a blockage of autophagic flow in neurons (Figure S6e,f, Supporting Information). Autophagic dysregulation and faulty autolysosome acidification in neurons have been shown to accumulate large numbers of Aβ‐positive autophagic vesicles, forming flower‐like perikaryal rosettes, which are a major source of senile plaques.^[^ ^44^ ^]^ These results suggest that E 171 exposure in neurons contributes to lysosomal underacidification through aberrant RyR‐Ca^2+^ release, subsequently inhibiting autophagic clearance and increasing AD‐related pathogenic protein accumulation.

Notably, pharmacological inhibition of RyR‐mediated Ca^2+^ release has consistently ameliorated both cognitive deficits and molecular pathology in AD models.^[^ ^12^, ^45^, ^46^ ^]^ Chronic dantrolene treatment (from 2 to 13 months of age) in 3xTg‐AD mice improved Morris Water Maze performance and reduced hippocampal Aβ burden.^[^ ^45^ ^]^ A 4‐week dantrolene regimen in presenilin‐mutant mice normalized endoplasmic reticulum Ca^2+^ dynamics, restored RyR2 expression, rescued synaptic transmission and plasticity, and attenuated cortical and hippocampal Aβ deposition.^[^ ^46^ ^]^ Moreover, a 30‐day Ryanodex course in 3xTg‐AD mice reinstated lysosomal vATPase V0a1 and V1B2 levels and prevented early pTau(S262) accumulation.^[^ ^12^ ^]^ In conjunction with our findings, RyR antagonism may serve as a potential therapeutic strategy to ameliorate both behavioral deficits and hallmark AD pathologies induced by environmental risk factors.

### 2.5 E 551 and Ag‐NPs Trigger AD‐Like Pathological Changes In Vitro and In Vivo via Aberrant Ca^2+^ Release‐Mediated Autophagy Defects

Beyond E 171, various nanoparticles, including E 551 and Ag‐NPs, are widely used in the food industry. Surprisingly, nanosilica and nanosilver trigger elevated intracellular Ca^2+^ signaling and are both neurotoxic.^[^ ^47^, ^48^, ^49^ ^]^ Therefore, we speculated that E 551 and Ag‐NPs may produce toxic effects in neurons common to those of E 171, such as abnormal lysosomal function and autophagic flow blockage through aberrant Ca^2+^ signaling.

**Figure 5:**
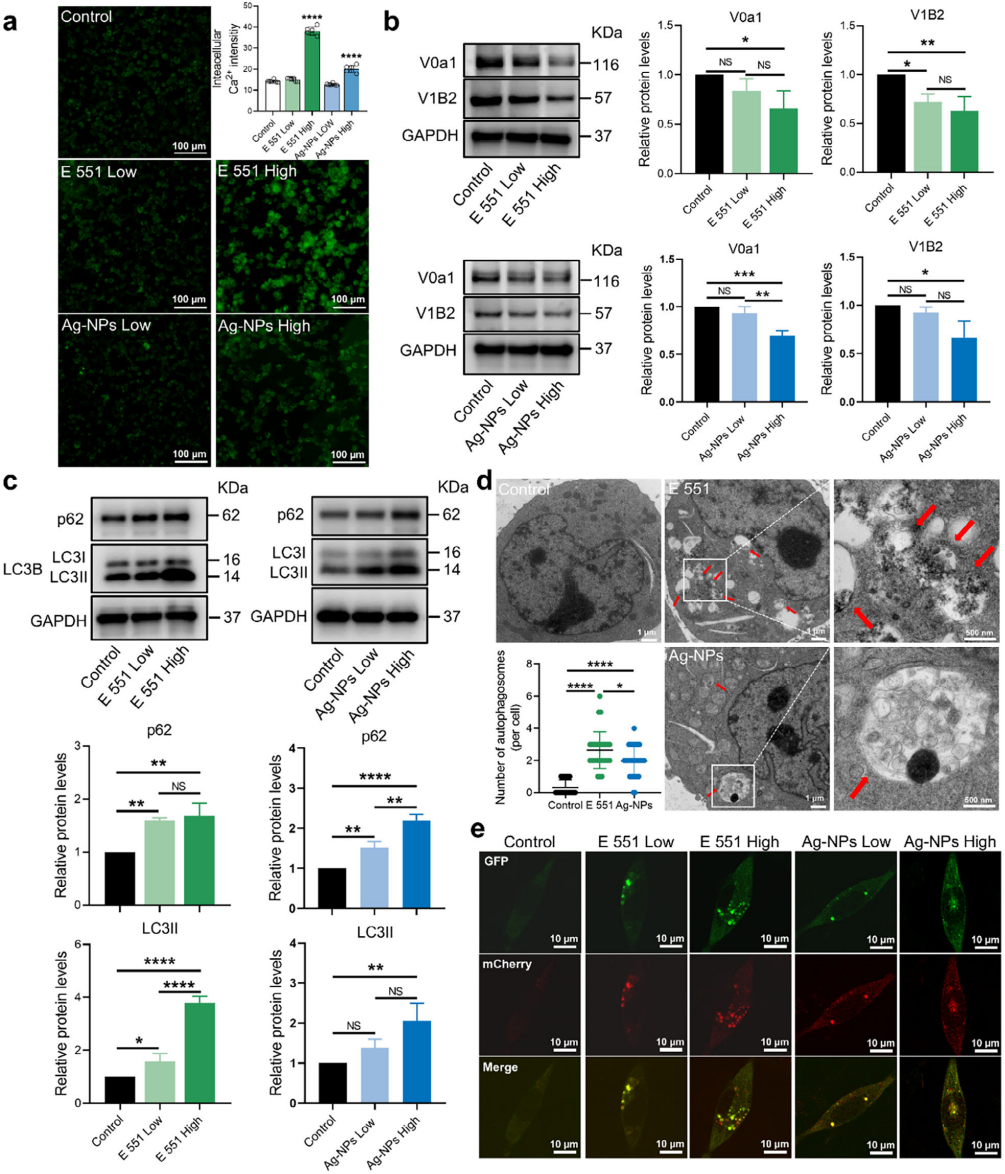
E 551 and Ag‐NPs trigger autophagy defects in HT22 cells through vATPase subunits deficiency caused by increased RyR‐Ca^2+^ signaling. a) Representative images of Ca^2+^ level in HT22 cells after E 551 (20 or 200 µg mL^−1^) or Ag‐NPs (1.5 or 15 µg mL^−1^) exposure. Statistical analysis was performed through one‐way ANOVA followed by Dunnett's test. b) Changes in protein expression of V0a1 and V1B2 in HT22 cells exposed to E 551 or Ag‐NPs. Statistical analysis was performed through one‐way ANOVA with Tukey's multiple comparison tests. c) Changes in protein expression of p62 and LC3B in HT22 cells exposed to E 551 or Ag‐NPs. Statistical analysis was performed through one‐way ANOVA with Tukey's multiple comparison tests. d) Representative images of HT22 cells obtained by TEM after exposure to high‐dose E 551 or Ag‐NPs (28 cells for each group). Autophagic vesicles are indicated by red arrows. Statistical analysis was performed through two‐tailed Mann–Whitney *U* tests. e) Representative images of Ad‐mCherry‐GFP transfected HT22 cells exposed to E 551 or Ag‐NPs. In the merged picture, yellow fluorescent puncta represent autophagosomes and red puncta represent autolysosomes. ^*^ *p* < 0.05, ^**^ *p* < 0.01, ^***^ *p* < 0.001, and ^****^ *p* < 0.0001.

**Figure 6:**
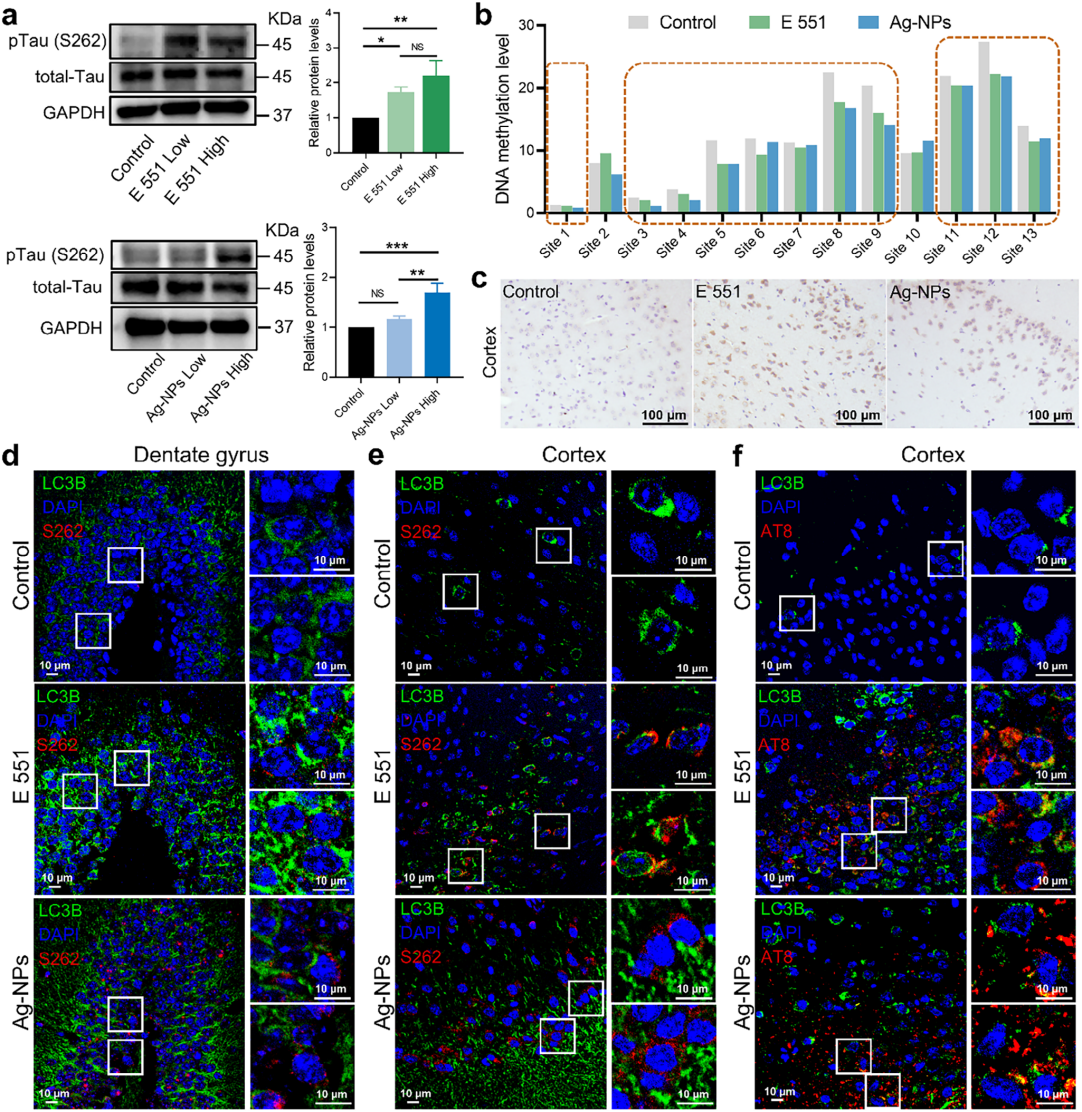
E 551 and Ag‐NPs trigger AD‐associated pathogenic protein accumulation in HT22 cells and mouse brains. a) Changes in protein expression of pTau(S262) and total‐Tau in HT22 cell. b) Changes in DNA methylation levels in the *RyR3* promoter region in the brain of mice exposed to E 551 or Ag‐NPs for 84 days. c) Representative immunohistochemistry images of Aβ 1–42 in the cortex of mice exposed to E 551 or Ag‐NPs for 84 days. d) Representative immunofluorescence images of LC3B and pTau(S262) in the brain's dentate gyrus region of mice exposed to E 551 or Ag‐NPs for 84 days. e) Representative immunofluorescence images of LC3B and pTau(S262) in the brain's cerebral cortex region of mice exposed to E 551 or Ag‐NPs for 84 days. f) Representative immunofluorescence images of LC3B and pTau(AT8) in the brain's cerebral cortex region of mice exposed to E 551 or Ag‐NPs for 84 days. Statistical analysis was performed through one‐way ANOVA with Tukey's multiple comparison tests, ^*^ *p* < 0.05, ^**^ *p* < 0.01, and ^***^ *p* < 0.001.

Detailed characterization data for E 551 have been reported in our previous study.^[^ ^16^ ^]^ Characterization of Ag‐NPs by SEM revealed dispersed spherical particles with a size distribution predominantly in the range of 10–20 nm (Figure S7a, Supporting Information). E 551 and Ag‐NPs exposed to HT22 cells were prepared following the generic Nanogenotox dispersion scheme and previous studies.^[^ ^43^, ^50^ ^]^ The hydrodynamic diameters and zeta potentials of E 551 and Ag‐NPs in the complete culture medium are shown in Table S2 (Supporting Information). HT22 cells were first exposed to E 551 (20 and 200 µg mL^−1^) and Ag‐NPs (1.5 and 15 µg mL^−1^) for 24 h. To determine appropriate dosing, HT22 cell viability was assessed following 24‐h exposure to E 551 and Ag‐NPs. The selected low and high concentrations corresponded to non‐cytotoxic (≥ 85% cell viability) and sub‐toxic levels (60–85% viability), respectively (Figure S7b, Supporting Information). SEM combined with EDS analysis confirmed the presence of SiO_2_ and Ag nanoparticles within HT22 cells after exposure to E 551 or Ag‐NPs (Figure S7c,d, Supporting Information). Moreover, Ca^2+^ quantification revealed that high doses of both E 551 (200 µg mL^−1^) and Ag‐NPs (15 µg mL^−1^) significantly increased Ca^2+^ levels in HT22 cells (**Figure** **5**a). Correspondingly, detection of vATPase subunits revealed that V0a1 and V1B2 expression levels significantly decreased after exposure to high‐dose E 551 and Ag‐NPs, thereby indicating lysosomal impairment (Figure 5b). Results from the LysoSensor Green and LysoTracker Red assays revealed that exposure to Ag‐NPs elicited effects similar to those induced by E 171, including a significant decrease in LysoSensor fluorescence intensity and a reduction in the number of LysoTracker Red puncta in HT22 cells (Figure S7e,f, Supporting Information). Notably, the number of LysoTracker Red puncta exhibited the most pronounced reduction in HT22 cells exposed to E 551, with the high‐dose E 551 group exhibiting only about one‐tenth of the puncta observed in the control group (Figure S7e,f, Supporting Information). This marked alteration is likely attributable to the localization of nanosilica within lysosomes after cellular uptake,^[^ ^51^, ^52^ ^]^ together with the abnormal enlargement of lysosomes induced by nanosilica exposure.^[^ ^53^ ^]^ Moreover, a slight increase in LysoSensor Green fluorescence intensity was observed (Figure S7e,f, Supporting Information), which might be attributable to the enlarged lysosomes.^[^ ^53^ ^]^ These findings indicate that E 551 and Ag‐NPs, similar to E 171, also cause marked lysosomal damage, which may subsequently trigger autophagic dysfunction. Analysis of p62 and LC3B expression revealed that both E 551 and Ag‐NPs exposure significantly increased p62 and LC3II levels in HT22 cells (Figure 5c). TEM of HT22 cells exposed to high‐dose E 551 and Ag‐NPs revealed a significant increase in autophagosome numbers (Figure 5d). In addition, assessment of autophagic flux demonstrated that exposure to E 551 and Ag‐NPs significantly increased the number of autophagosomes and markedly reduced the number of autolysosomes, thus indicating that both substances disrupt the normal autophagic process (Figure 5e; Figure S7g,h, Supporting Information). More importantly, examination of HT22 cells after exposure to E 551 and Ag‐NPs revealed that both food‐grade nanomaterials triggered autophagy impairment, leading to pTau(S262) accumulation in neurons (**Figure** **6**a). These results suggest that E 551 and Ag‐NPs share toxic effects with E 171, specifically, impairing the autophagy process triggered by aberrant Ca^2+^ signaling, thereby resulting in pTau accumulation.

Mice were exposed to E 551 (1000 mg kg^−1^ bw per day) or Ag‐NPs (0.35 mg kg^−1^ bw per day) for 84 days via oral feeding or gavage, respectively, to assess the potential in vivo effects of E 551 and Ag‐NPs exposure. The exposure dose was set at twice the highest estimated human exposure from food (50 mg kg^−1^ bw per day)^[^ ^54^ ^]^ for E 551 and five times the supplier's recommended daily intake (0.006 mg kg^−1^ bw per day) for Ag‐NPs, and these were both normalized to the body surface area.^[^ ^19^ ^]^ Bisulfite‐pyrosequencing methylation analysis of the *RyR3* promoter revealed reduced methylation levels at 11 and 12 out of 13 examined CpG sites in the mouse brain following E 551 and Ag‐NPs subchronic exposure, respectively (Figure 6b). Reduced methylation in the *RyR3* promoter region increased *RyR3* gene expression levels (Figure S7i, Supporting Information). Immunohistochemical detection of Aβ 1–42 was performed on the brains of the two food‐grade nanoparticle exposure groups. The results showed that both E 551 and Ag‐NPs exposures resulted in Aβ 1–42 deposition in mouse brains (Figure 6c). We further focused on changes in the expression of pTau (S262 and AT8) and the autophagy marker LC3B in the cerebral cortex and hippocampus. The results showed that LC3B staining was considerably increased in the E 551‐ and Ag‐NPs‐exposed groups, and both showed significant pTau (S262 and AT8) foci (Figure 6d–f).

In this study, the AD risk associated with three food‐grade nanoparticles and their potential mechanisms were investigated through a combination of oral exposure in mice and in vitro exposure using HT22 cells. HT22 cells, derived from murine hippocampal neurons, have proven to be a sensitive and functionally relevant model for studying AD‐related neurotoxicity. Multiple studies have demonstrated that HT22 cells faithfully mimic core pathological mechanisms observed in vivo, including Aβ accumulation, inflammatory responses, mitochondrial dysfunction, and oxidative stress.^[^ ^55^, ^56^, ^57^, ^58^ ^]^ For instance, the molecular profiles observed in Aβ‐treated HT22 cells closely mirror those found in postmortem AD brain specimens.^[^ ^55^ ^]^ In addition, HT22 cells have been used to investigate the accumulation of Aβ, excessive phosphorylation of Tau, and inflammatory responses induced by environmental neurotoxins.^[^ ^56^ ^]^ They have also been employed to examine the restorative effects of drug treatments on Aβ‐induced mitochondrial dysfunction.^[^ ^57^, ^58^ ^]^ Along with our findings, these responses collectively validate HT22 cells as a reliable proxy for hippocampal neurons in vivo, thereby enabling reproducible investigation of AD‐relevant mechanisms and therapeutic efficacy. This study employed two administration routes, the premixed feed method and the gavage method, to investigate the toxicological characteristics and mechanisms of action of food‐grade nanoparticles. The premixed feed method preserves the interaction between the food matrix and nanoparticles, thereby providing a more accurate simulation of human dietary exposure to the food additives E 171 and E 551.^[^ ^59^ ^]^ In contrast, the gavage method for administering Ag‐NPs simulates direct through‐liquid‐phase oral supplementation, thereby accurately replicating real‐world exposure conditions. Liquid‐phase exposure during digestion transits more rapidly through the gastrointestinal tract compared to solid food, potentially influencing absorption dynamics.^[^ ^60^ ^]^ Given that different administration routes can result in distinct absorption and distribution profiles in vivo,^[^ ^61^ ^]^ it is essential to select an exposure model that most accurately reflects human exposure scenarios.

The present study investigated two key hippocampal subfields associated with AD pathology: CA1 and the dentate gyrus. The CA1 region is consistently recognized as the earliest site of neuronal loss and is particularly susceptible to amyloid and tau aggregation.^[^ ^62^, ^63^ ^]^ Accordingly, our findings revealed pronounced nuclear pyknosis, Aβ deposition, and pTau‐positive staining in the CA1 region following exposure to food‐grade nanoparticles, which underscore its pivotal role in AD‐like neuropathological alterations. The dentate gyrus plays a critical role in adult hippocampal neurogenesis and is a principal site for pTau propagation from the entorhinal cortex; in AD patients, excessive pTau accumulation in the dentate gyrus correlates with impaired neurogenesis and cognitive decline.^[^ ^64^, ^65^ ^]^ In this study, food‐grade nanoparticle exposure led to the simultaneous accumulation of pTau protein and LC3B in the dentate gyrus region, thereby suggesting a potentially important role of autophagy in mediating these pathological changes.

Beyond Aβ and tau pathology, AD also involves nuclear alterations that contribute to disease progression.^[^ ^66^ ^]^ Among these nuclear alterations, aberrant epigenetic alterations, including histone post‐translational modifications and DNA methylation, have been increasingly implicated in AD pathogenesis.^[^ ^67^, ^68^ ^]^ In the present study, exposure to food‐grade nanoparticles induced aberrant DNA methylation accompanied by lysosomal impairment, defective autophagic flux, and the accumulation of Aβ and pTau in neurons. These findings not only corroborate previous reports of nuclear dysfunction in AD but also extend them by identifying nanoparticle‐induced epigenetic alterations as a convergent mechanism linking nanoparticle exposure to AD‐like pathology.

Autophagy is an intracellular catabolic process essential for cytoplasmic quality control.^[^ ^69^ ^]^ Its neuroprotective effect arises from its ability to eliminate pathogenic proteins (e.g., Aβ, α‐synuclein, or pTau), and dysfunctional autophagy is closely associated with neurodegenerative diseases.^[^ ^70^ ^]^ In our study, the three food‐grade nanoparticles impaired autophagy and hindered the clearance of AD‐associated pathogenic proteins both in vivo and in vitro. Notably, a few non‐food‐grade engineered nanoparticles have been found to influence the autophagy process.^[^ ^13^ ^]^ While this property enhances the potential applications of nanomedicine, it also raises concerns about the risk of nanoparticle exposure leading to the accumulation of pathogenic proteins through interference with autophagic pathways. This highlights the need for further research on the role of nanoparticle exposure in autophagy‐associated neurodegenerative diseases.

Nonetheless, a limitation of this study is the exclusive use of male mice. This choice aimed to minimize variability arising from the female estrous cycle, thereby maintaining more consistent endocrine and metabolic baselines. However, biological sex may influence both the severity and underlying mechanisms of E 171‐induced toxicity. Consequently, our findings may not fully capture potential female‐specific effects. Future investigations will incorporate both male and female cohorts to delineate sex‐dependent toxicological profiles.

## 3 Conclusion

This study established an epigenetic link between food‐grade nanoparticle exposure and autophagy defects, demonstrating that three food‐grade nanoparticles—E 171, E 551, and Ag‐NPs—share a common toxic effect: triggering AD‐like pathology by impairing autophagy in neurons. Mechanistically, nanoparticle‐induced epigenetic alterations and subsequent intracellular Ca^2+^ abnormalities lead to impaired lysosomal function and autophagy. This impaired autophagic clearance process results in the accumulation of AD‐related proteins (Aβ and pTau) in neurons. Since the intake of nanoparticles in food is frequent and difficult to detect, most populations may be at risk of exposure. More importantly, nanoparticles have a wide range of modulatory effects on autophagy, which suggests that more nanoparticles may exist that produce similar toxic effects.^[^ ^13^ ^]^ Three types of food‐grade hard nanoparticles, namely metal oxide, metal, and ceramic nanoparticles, were selected to reveal the potential AD risk of individual types of food‐grade nanoparticles. However, the risks arising from co‐exposure to these nanoparticles may be more complex, as humans are often exposed to a mixture of these nanoparticles in food. Additionally, soft nanoparticles such as polystyrene have also been found to promote the development of neurodegenerative diseases, including PD and ALS.^[^ ^33^, ^34^ ^]^ Therefore, there is a need to investigate the broader links and mechanisms between nanoparticles and neurodegenerative diseases. In summary, our findings provide new evidence and perspectives on how food‐grade nanoparticles can trigger or exacerbate neurodegenerative diseases.

## 4 Experimental Section

### Physicochemical Characterization

E 171‐1, E 171‐2, E 551, and Ag‐NPs were obtained from Jiang Hu Titanium White Product Co., Ltd. (Shanghai, China), Merck KGaA. (Darmstadt, Germany), Anji Donglai Pharmaceutical Excipient Co., Ltd. (Huzhou, China), and Natural Path Silver Wings (TN, USA), respectively. The detailed characterization data for E 171 and E 551 were previously published in the earlier studies.^[^ ^16^, ^18^ ^]^ The Ag‐NPs were visualized under a Nova Nano 450 SEM (Thermo Fisher Scientific) in TEM mode. Hydrodynamic diameters and zeta potentials in complete culture medium were determined via dynamic and electrophoretic light scattering using a Zetasizer Nano‐ZS90 (Malvern Instruments, Worcestershire, UK).

### Animal Experiments

Male BALB/c mice (18–22 g), a strain commonly utilized in nanotoxicological evaluations,^[^ ^71^, ^72^ ^]^ were provided by the Beijing Vital River Laboratory Animal Technologies Co., Ltd (Beijing, China). After acclimating for 7 days, mice were housed under a 12‐h light/dark cycle at 25 ± 1 °C with 50 ± 10% relative humidity. Food and water were provided ad libitum. All procedures complied with the Animal Care and Use Committee of Zhejiang University School of Medicine (ZJU20230346) and followed the guidelines for the Use of Animals in Toxicology.

Mice (*n* = 8; *n* indicates the number of mice in each group) were exposed to low, middle, and high doses (8, 80, and 320 mg kg^−1^ bw per day, respectively) of the two E 171 types via premixed feed and fed continuously for 28 or 84 days. The murine‐equivalent doses were calculated based on the mean daily dietary intake for adults aged 18–64 years (0.6–6.8 mg kg^−1^ bw) as estimated by the EFSA, and corresponded to 8 and 80 mg kg^−1^ bw in mice following body surface area conversion (0.6 × 12.3 ≈ 8, 6.8 × 12.3 ≈ 80 mg kg^−1^ bw).^[^ ^14^, ^19^ ^]^ At the 95th percentile, adult exposure estimates ranged from 2.2 to 15 mg kg^−1^, which translated to a murine equivalent of up to 185 mg kg^−1^ (15 × 12.3 ≈ 185 mg kg^−1^).^[^ ^14^, ^19^ ^]^ A high dose of 320 mg kg^−1^ bw was included to account for additional sources of exposure such as pharmaceuticals and cosmetics.^[^ ^19^ ^]^ The exposure durations of 28 and 84 days were selected in accordance with the Organization for Economic Co‐operation and Development Test Guidelines 407 and 408, respectively. This two‐phase design enables the detection of progressive toxicity and provides insight into time‐dependent changes in toxicological responses. Mice exposed to E 551 (1000 mg kg^−1^ bw per day) and Ag‐NPs (0.35 mg kg^−1^ bw per day) via premixed feed and gavage, respectively, were continuously exposed for 84 days. According to the EFSA, the maximum estimated daily exposure to E 551 from food sources is ≈50 mg kg^−1^ bw.^[^ ^54^ ^]^ This means that a human intake of 50 mg kg^−1^ is equivalent to 615 mg kg^−1^ bw in mice, based on body surface area conversion (50 × 12.3 = 615 mg kg^−1^ bw).^[^ ^19^ ^]^ However, dietary intake is not the sole exposure pathway; E 551 is also commonly present in dietary supplements, pharmaceuticals, and cosmetics, and potentially reaches up to 25 mg kg^−1^ bw through supplemental intake.^[^ ^73^ ^]^ This suggests that the total daily intake of E 551 in humans may reach 75 mg kg^−1^ bw, corresponding to 922.5 mg kg^−1^ bw in mice, calculated using body surface area conversion (75 × 12.3 = 922.5 mg kg^−1^ bw).^[^ ^19^ ^]^ Accordingly, the exposure dose of E 551 applied in this study was set at 1000 mg kg^−1^ bw. For Ag‐NPs, long‐term supplement use in adults (0.006 mg kg^−1^ bw) translates to 0.07 mg kg^−1^ bw in mice by the same scaling (0.006 × 12.3 ≈ 0.07 mg kg^−1^ bw). In addition, short‐term recommended dose (0.042 mg kg^−1^ bw, 14 days) translates to 0.52 mg kg^−1^ bw in mice by the same scaling (0.042 × 12.3 ≈ 0.52 mg kg^−1^ bw). Moreover, dietary supplements represent only one exposure route, and Ag‐NPs appear in nearly 450 consumer products (including food‐contact materials, textiles, toiletries, and cosmetics).^[^ ^74^ ^]^ Considering both the long‐term and short‐term recommended doses, as well as the likelihood of multi‐route exposure, the Ag‐NP dose administered to mice in this study was set at 0.35 mg kg^−1^ bw, falling between 0.07 and 0.52 mg kg^−1^ bw. Feeds containing E 171‐1, E 171‐2, or E 551 were prepared by Shanghai Puluteng Biotechnology Co., Ltd. (Shanghai, China). To incorporate E 171 or E 551 into the animal feed, the respective powder was gradually blended with equal portions of feed ingredients until the desired concentration was reached. The resulting premix was subsequently processed using a VH‐50 high‐efficiency mixer (Tian He Machinery Equipment, Shanghai, China), during which oils, water, and fats were incrementally added. The prepared feed was irradiated with Co‐60 at a dose of 10 kGy. This dose was commonly employed in animal feed processing and did not significantly affect the physicochemical properties of anatase TiO_2_, which is the crystal structure of both E 171‐1 and E 171‐2.^[^ ^75^, ^76^ ^]^ The consistency of Ti content in the high‐dose E 171 feed samples was confirmed using inductively coupled plasma‐Mass Spectrometry (Figure S1b, Supporting Information), with the specific methodology provided in the Supporting Information.

### H&E, Immunohistochemistry, TUNEL, and Immunofluorescence

H&E, immunohistochemistry, TUNEL, and immunofluorescence were carried out on 4‐µm‐thick sections of formalin‐fixed, paraffin‐embedded tissue samples from coated slides. Immunohistochemical and immunofluorescence analyses were performed using standard procedures. Coronal brain sections were pretreated with heat‐mediated antigen retrieval using sodium citrate buffer (pH 6, Epitope Retrieval Solution 1) for 20 min. Subsequently, sections were incubated at 25 °C with Aβ 1–42 antibody (Abcam, Cambridge, UK) for 15 min and detected using a horseradish peroxidase (HRP) conjugated compact polymer system. The antibodies used for immunofluorescence were LC3B (Abcam), pTau(S262) (Thermo Fisher Scientific, MA, USA), and pTau(AT8) (Thermo Fisher Scientific). Immunofluorescence was observed and imaged using a TCS SP8 X confocal microscope (Leica, Wetzlar, Germany).

### SEM, EDS, and TEM Analyses

After subchronic exposure, samples from the cortex and hippocampus were fixed overnight in 2.5% (v/v) glutaraldehyde. After washing and dehydration, the samples were cut into 110–130 nm flakes and collected on copper or nickel grids. Subsequently, they were then observed using a Nova Nano 450 SEM (Thermo Fisher Scientific) in TEM mode, and EDS was performed using an EDAX TEAM Octane EDS‐70 (Ametek, PA, USA). A Talos L120C TEM (Thermo Fisher Scientific) was used to detect autophagic changes in HT22 cells.

### Global DNA Methylation Analysis

Genomic DNA was extracted from mouse brains using the Wizard Genomic DNA Purification Kit (Promega, Madison, WI, USA). DNA integrity was confirmed by 1% agarose gel electrophoresis. DNA purity and concentration were measured using a NanoDrop 2000 spectrophotometer (Thermo Fisher Scientific), with A260/A280 ratios between 1.8 and 2.0. For each group, equal amounts of DNA from individual samples were pooled to generate a representative composite sample, which was then diluted to a final concentration of 50 ng µL^−1^. The levels of 5‐mC and 5‐hmC were detected using MethylFlash Global DNA Methylation (5‐mC) and hydroxymethylation (5‐hmC) ELISA Easy kits (EpiGentek, USA).

### WGBS

Genomic DNA was extracted from brain tissue using the DNeasy Blood & Tissue Kit (Qiagen) and assessed for integrity on an Agilent 2100 Bioanalyzer. Samples exhibiting a single primary band >10 kb with no signs of degradation or contamination were considered qualified. Each 3 µg DNA sample was spiked with 26 ng unmethylated λDNA and fragmented to 200–300 bp by sonication. Fragmented DNA underwent end repair, A‐tailing, and cytosine‐methylated adapter ligation, and then was bisulfite‐converted twice using the EZ DNA Methylation‐Gold Kit (Zymo Research, CA, USA). Libraries were PCR‐amplified with HiFi HotStart Uracil + ReadyMix (2×), quantified on a Qubit 2.0 Fluorometer, and size‐verified on the Agilent 2100. Paired‐end reads were generated by Shanghai Biotechnology (Shanghai, China). Raw reads were filtered with fastp v0.20.0 (Q20 ≥ 90%) and trimmed of adapters and low‐quality bases using Trim Galore v0.4.1. Clean reads were aligned to the mouse reference genome (mm10) with Bismark v0.15.0 (Bowtie2 v2.2.9); PCR duplicates were then removed. Genome‐wide methylation calls were extracted using Bison v0.4.0, and methylation levels and coverage were calculated in 100 kb windows to generate the methylome profiles.

### Bisulfite‐Pyrosequencing Analysis

The bisulfite‐pyrosequencing methylation analysis of the promoter of *RyR3* was performed based on the previous study.^[^ ^16^ ^]^ Briefly, DNA was extracted from the brains of three randomly selected mice in each of the Control and food‐grade nanoparticle‐exposed groups (E 171‐2 middle, E 551, and Ag‐NPs) and pooled in equal amounts as representative samples. Bisulfite‐converted DNA was PCR‐amplified using *RyR3*‐specific primers (forward: 5′‐GGGAGTTGGGTTAATATTTGATTG‐3′; reverse: 5′‐ACCCCAATCCCTAACTCTACCC‐3′). Following PCR product purification, pyrosequencing was conducted using the PyroMark Q96 ID system (Qiagen) according to the manufacturer's instructions. The primer for the pyrosequencing was 5′‐CCCTAACTCTACCCC‐3′. DNA methylation levels were quantified at 13 CpG sites.

### Western Blotting

Samples from mouse brains or HT22 cells were lysed using lysis buffer (100 mm NaCl, 1 mm EDTA, 10 mm Tris‐HCl, 0.5% Triton X‐100, 10% glycerol, 1 mm Na_3_VO_4_, 1 mm PMSF, and 5 mm NaF). Protein concentrations were determined using the Quick Start Bradford protein Assay Kit (Bio‐Rad Laboratories, CA, USA). Equal proteins were separated by 10% SDS‐PAGE, 12% SDS‐PAGE, or 3−8% NuPAGE (Thermo Fisher Scientific) and transferred to polyvinylidene fluoride membranes. The membranes were blocked in 5% nonfat powdered milk and incubated overnight at 4 °C with the following primary antibodies: RyR3 (1:1000, AF4611, Affinity Biosciences, OH, USA), FEV (1:1000, AP21664a, Abcepta, SD, USA), β‐actin (1:1000, AF5003, Beyotime Biotechnology, Shanghai, China), p62 (1:1000, AG4400, Beyotime Biotechnology), LC3B (1:1000, AL221, Beyotime Biotechnology), GAPDH (1:1000, AF1186, Beyotime Biotechnology), pTau(S262) (1:500, 44–750G, Thermo Fisher Scientific), total‐Tau (1:500, 46687, Cell Signaling Technology, MA, USA), ATP6V1B2 (1:1000, 15097‐1‐AP, Proteintech, Wuhan, China), and ATP6V0a1 (1:1000, 13828‐1‐AP, Proteintech). Blots were incubated with horseradish peroxidase‐conjugated secondary antibodies (1:5000, A0208, Beyotime Biotechnology). Semi‐quantitative analysis of relative protein expression levels was conducted using densitometry with Image Lab version 5.2. Background‐subtracted target signals were normalized to the corresponding β‐actin or GAPDH band in each lane, which served as stable loading controls for brain tissue and HT22 cells, respectively. All experiments were carried out in triplicate.

### Behavioral Tests

Prior to completion of the subchronic exposure period, male mice (18–20 weeks old) underwent behavioral testing in the Y‐maze, NOR, and Morris water maze.

### Y‐Maze

The Y‐maze had three identical arms; each mouse was placed at the end of the starting arm and allowed to explore freely for 5 min. All behaviors were video recorded using a camera mounted above the maze and were assessed using automated video tracking (ANY‐maze). Spontaneous alternations were defined as consecutive entries into each arm without repetition. The percentage of spontaneous alteration was calculated using the following equation:

(1) $$\begin{array}{ccc} \mathrm{Spontaneous}\;\mathrm{alternation}\% & = & \frac{\mathrm{the}\;\mathrm{number}\;\mathrm{of}\;\mathrm{actual}\;\mathrm{alternations}}{\mathrm{the}\;\mathrm{number}\;\mathrm{of}\;\mathrm{total}\;\mathrm{arm}\;\mathrm{entries}\;-\;2}\\ & & \;\times \;100\% \end{array}$$

### Morris Water Maze

Water maze testing was performed as previously outlined.^[^ ^77^ ^]^ On the training day, a platform was placed at the center of the platform quadrant. After 4 days of training, the platform was removed for the probing experiment. The mice started from the quadrant furthest from the previous platform. Their movements were recorded using a video camera and analyzed using Water Maze software (Actimetrics, IL, USA). Quadrant time was calculated using the following equation:

(2) $$\begin{array}{ccc} \mathrm{Quadrant}\;\mathrm{time}\% & = & \frac{\mathrm{the}\;\mathrm{time}\;\mathrm{spent}\;\mathrm{in}\;\mathrm{the}\;\mathrm{platform}\;\mathrm{quadrant}}{\mathrm{the}\;\mathrm{total}\;\mathrm{time}}\\ & & \;\times \;100\% \end{array}$$

### NOR

NOR was performed according to the method reported in a previous study.^[^ ^78^ ^]^ Briefly, during the training phase, two identical cylindrical rod objects (familiar objects) were placed in the test arena, and each mouse was allowed to explore freely for 5 min. Object exploration behavior was recorded using automated video tracking (ANY‐maze). Nose‐tip contact within 2 cm of an object was defined as effective exploration. After a 24‐h interval, one of the cylindrical rods was replaced with a rectangular block of the same material (novel object), and the mouse was reintroduced into the test arena for the testing phase. The discrimination index was calculated as follows:

(3) $$\begin{array}{ccc} \mathrm{Discrimination}\;\mathrm{index}\% & = & \frac{\mathrm{the}\;\mathrm{time}\;\mathrm{spent}\;\mathrm{exploring}\;\mathrm{the}\;\mathrm{new}\;\mathrm{object}}{\mathrm{the}\;\mathrm{total}\;\mathrm{time}\;\mathrm{spent}\;\mathrm{exploring}\;\mathrm{both}\;\mathrm{objects}}\\ & & \;\times \;100\% \end{array}$$

### Cell Culture

HT22 cells were purchased from the Chinese Academy of Sciences, Typical Culture Collection Committee Cell Bank (Shanghai, China). Cells were cultured in high‐glucose Dulbecco's modified Eagle's medium (DMEM, Gibco, ThermoFisher Scientific) supplemented with 100 U mL^−1^ penicillin, 100 µg mL^−1^ streptomycin, and 10% fetal bovine serum (Gibco, ThermoFisher Scientific), which collectively constituted the complete culture medium. Cultures were maintained at 37 °C and 5% CO_2_.

### Food‐Grade Nanoparticle Dispersion

E 171 and E 551 dispersions for cell exposure were prepared according to the generic Nanogenotox dispersion scheme described by Jensen et al.^[^ ^43^ ^]^ Briefly, a stock suspension of 2.56 mg mL^−1^ was first prepared by dispersing E 171 or E 551 powder in 0.05% (w/v) bovine serum albumin solution and probe‐sonicated for 16 min at 400 W on ice. Suspensions were freshly prepared before each experiment and diluted to the target concentrations in complete culture medium. Ag‐NPs were obtained at a stock concentration of 500 µg mL^−1^ and prepared according to previous studies.^[^ ^50^ ^]^

### Ca^2+^ Detection

Ca^2+^ was detected by labeling with the calcium indicator Fluo‐4 AM (Thermo Fisher Scientific). Imaging and image acquisition were performed using an ImageXpress Micrao Contocal (Molecular Devices, CA, USA), and fluorescence intensity was quantified using ImageJ 1.54f software.

### mCherry‐GFP‐LC3B Transient Transfection

To assess autophagic flux, HT22 cells were infected with an adenovirus expressing the mCherry‐GFP‐LC3B fusion protein (MOI = 100, Vigene Bioscience, Jinan, China). After 12 h of infection, the medium was replaced with DMEM complete medium. Transfection efficiency (>90%, Figure S6b, Supporting Information), determined from three independent experiments, was assessed by confocal microscopy 24 h post‐infection. Uninfected cells were used as the negative control. Subsequently, after 24 h of exposure to E 171, E 551, or Ag‐NPs, the cells were observed, and fluorescent photographs were obtained using a TCS SP8 X confocal microscope (Leica). GFP and mCherry were sequentially excited at 488 and 552 nm, respectively, using identical laser power and detector gain across all groups. Three random fields (≈30 cells per field) were captured for each of three biological replicates (*n* = 3; *n* represents three independent experiments). Autophagosomes were defined as co‐localized GFP^+^/mCherry^+^ (yellow) puncta, while autolysosomes were identified as mCherry^+^ (red) puncta only. Puncta counts were normalized to the number of cells per field.

### Statistical Analysis

The statistical analyses were performed using Prism 8.0 software (GraphPad). Normality was assessed for each dataset using the Shapiro–Wilk test, while homogeneity of variances was evaluated using the Brown–Forsythe test. One‐way ANOVA followed by Dunnett's test or Tukey's test was used to test for statistical differences between multiple groups. Two‐group comparisons were conducted using Student's t‐test (two‐tailed). In cases where normality or homogeneity of variances was violated, the parametric test was replaced with a non‐parametric alternative (two‐tailed Mann–Whitney *U* test for two‐group comparisons). Values are expressed as the mean ± standard deviation (SD). Statistical significance was defined as *P* values < 0.05. Each experiment was independently repeated at least three times.

## Conflict of Interest

The authors declare no conflict of interest.

## Supporting information



Supporting Information

## Data Availability

The data that support the findings of this study are available from the corresponding author upon reasonable request.
