# Supplementary material for: Oral Exposure to Food‐Grade Nanoparticles Poses a Risk of Alzheimer's Disease‐Like Symptoms by Triggering Autophagy Defects in Neurons
Source: Adv Sci (Weinh). 2025 Oct 27;13(3):e08096. doi: 10.1002/advs.202508096 (PMC12806370; doi:10.1002/advs.202508096)
Supplement: Supplementary file 1 — Supporting Information [file ADVS-13-e08096-s001.docx]

Supporting Information

Oral Exposure to Food-Grade Nanoparticles Poses a Risk of Alzheimer’s Disease-Like Symptoms by Triggering Autophagy Defects in Neurons

Jiaxin Shang, Jun Yan, He Lou, Xuanxi Jiang, Ziyue Wang, Yue Gao*, Xiaohui Fan*, and Xiaoyan Lu*

J. Shang, J. Yan, H. Lou, X. Jiang, Z. Wang, X. Fan, X. Lu

Pharmaceutical Informatics Institute, College of Pharmaceutical Sciences, Zhejiang University

Hangzhou 310058, China.

E-mail: fanxh@zju.edu.cn; luxy@zju.edu.cn

X. Fan, X. Lu

State Key Laboratory of Chinese Medicine Modernization, Innovation Center of Yangtze River Delta, Zhejiang University

Jiaxing, 314102, China.

X. Fan, X. Lu

Jinhua Institute of Zhejiang University

Jinhua, 321299, China.

Y. Gao

Department of Pharmaceutical Sciences, Beijing Institute of Radiation Medicine

Beijing 100850, China

E-mail: gaoyue@bmi.ac.cn

Author 1 and Author 2 contributed equally to this work.

**Experimental Section**

*Inductively Coupled Plasma-Mass Spectrometry (ICP-MS) Analysis*: Three feed samples used in the high-dose E 171-1 and E 171-2 exposure experiments were randomly selected from each group. From each feed sample, three subsamples (~0.06 g each) were collected from distinct locations within the batch, specifically, the edge, one-quarter, and central region (designated as positions 1, 2, and 3; **Figure S1b**), to evaluate the uniformity of TiO_2_ content. Each sample was digested by adding 1 mL of HF and 5 mL of HNO_3_ (65–68%), followed by a staged digestion protocol using a microwave-assisted system (MARS 6, CEM Corporation, Matthews, NC). The digestion process included sequential heating at 110 ℃ for 10 min and 180 ℃ for 20 min. After digestion, the vessels were held at 160 ℃ for 1 h to release residual acid vapors. The final volume was adjusted to 50 mL with ultrapure water before analysis. Finally, the TiO_2_ content of the solution was determined using an Elan DRC-e ICP-MS instrument (PerkinElmer, USA) and calculated using the following equation:

$$\text{TiO}\text{2}\text{ }\text{content (mg}\text{/}\text{kg)}\text{ }\text{=}\text{ }\frac{\text{c × v × }\text{79.87}}{\text{m}\text{ × }\text{47.87}}$$

where c (mg/L) is the Ti concentration in the feed, v (L) is the final volume of the sample solution after digestion, and m (kg) is the weight of the feed sample.

*Quantitative Reverse Transcription Polymerase Chain Reaction (**RT-qPCR) Analysis*: Total RNA was extracted from mouse brain tissue using the RNeasy Mini Kit (Qiagen, Germany) following the manufacturer’s protocol. RNA integrity was verified using the Qubit 4 (Thermo Fisher Scientific). RNA purity and concentration were assessed on a NanoDrop 2000 spectrophotometer (Thermo Fisher Scientific), with A260/A280 ratios of 1.8–2.0 considered acceptable. Equal amounts of RNA from each sample within a group were pooled to generate a representative composite sample, which was then used for cDNA synthesis with the HiFiScript cDNA Synthesis Kit (CWBIO, Taizhou, China). RT-qPCR was performed on a CFX96 Touch™ system (Bio-Rad, CA, USA) using Hieff UNICON^®^ qPCR SYBR Green Master Mix (Yeasen Biotech, Shanghai, China). The specific primers (Sangon Biotech, Shanghai, China) are shown in **Table S3**. Primer efficiencies (90–105%) were determined by five-point, 10-fold dilution series and confirmed by standard‐curve slopes. Melt‐curve analysis verified single amplicons. Expression levels were normalized to *Gapdh* and *Rps29*, and relative fold changes were calculated using the 2^-ΔΔCt^ method. All experiments were carried out in triplicate.

*Cell Viability Assay*: HT22 cells were harvested and seeded into 96-well plates at 100 μL per well of complete culture medium. Food-grade nanoparticle suspensions (see Experimental Section: *Food-Grade Nanoparticle Dispersion*) were serially diluted in complete culture medium to prepare exposure solutions of varying concentrations. After 24 h of incubation, each well was then treated with 100 μL of the respective nanoparticle solution, while blank control wells received an equal volume of complete culture medium without nanoparticles. Following 24 h of exposure, 100 μL of complete culture medium containing 10% CCK-8 reagent was added to each well. The plates were incubated for 1.5 h at 37 ℃ in the dark. Optical density (OD) was measured at 450 nm using a microplate reader (Infinite M1000 Pro, Tecan, Switzerland). Cell viability (%) was calculated using the following formula:

$$\text{Cell}\text{ }\text{viability}\text{ }\text{(\%)}\text{ }\text{=}\text{ }\frac{{\text{O}\text{D}}_{\text{exposure}}\text{ }\text{-}\text{ }{\text{O}\text{D}}_{\text{blank}}}{{\text{O}\text{D}}_{\text{control}}\text{ }\text{-}\text{ }{\text{O}\text{D}}_{\text{blank}}}\text{ × 100\%}$$

*LysoSensor and LysoTracker* *Analysis*: HT22 cells were exposed to E 171, E 551, or Ag-NPs for 24 h, followed by sequential staining with LysoSensor™ Green DND (1 μM, 30 min; Thermo Fisher Scientific) and LysoTracker™ Red (75 nM, 15 min; Thermo Fisher Scientific) under standard culture conditions. LysoSensor Green exhibits a pH-dependent increase in fluorescence intensity upon acidification, while LysoTracker Red is commonly used to label lysosomes based on their acidic environment. Cells were then examined, and fluorescence images were acquired using a TCS SP8 X confocal microscope (Leica). For each of the three biological replicates (*n* = 3), three randomly selected fields (~ 30 cells per field) were captured. LysoSensor fluorescence intensity and the number of LysoTracker puncta were quantified using ImageJ software (version 1.54f).


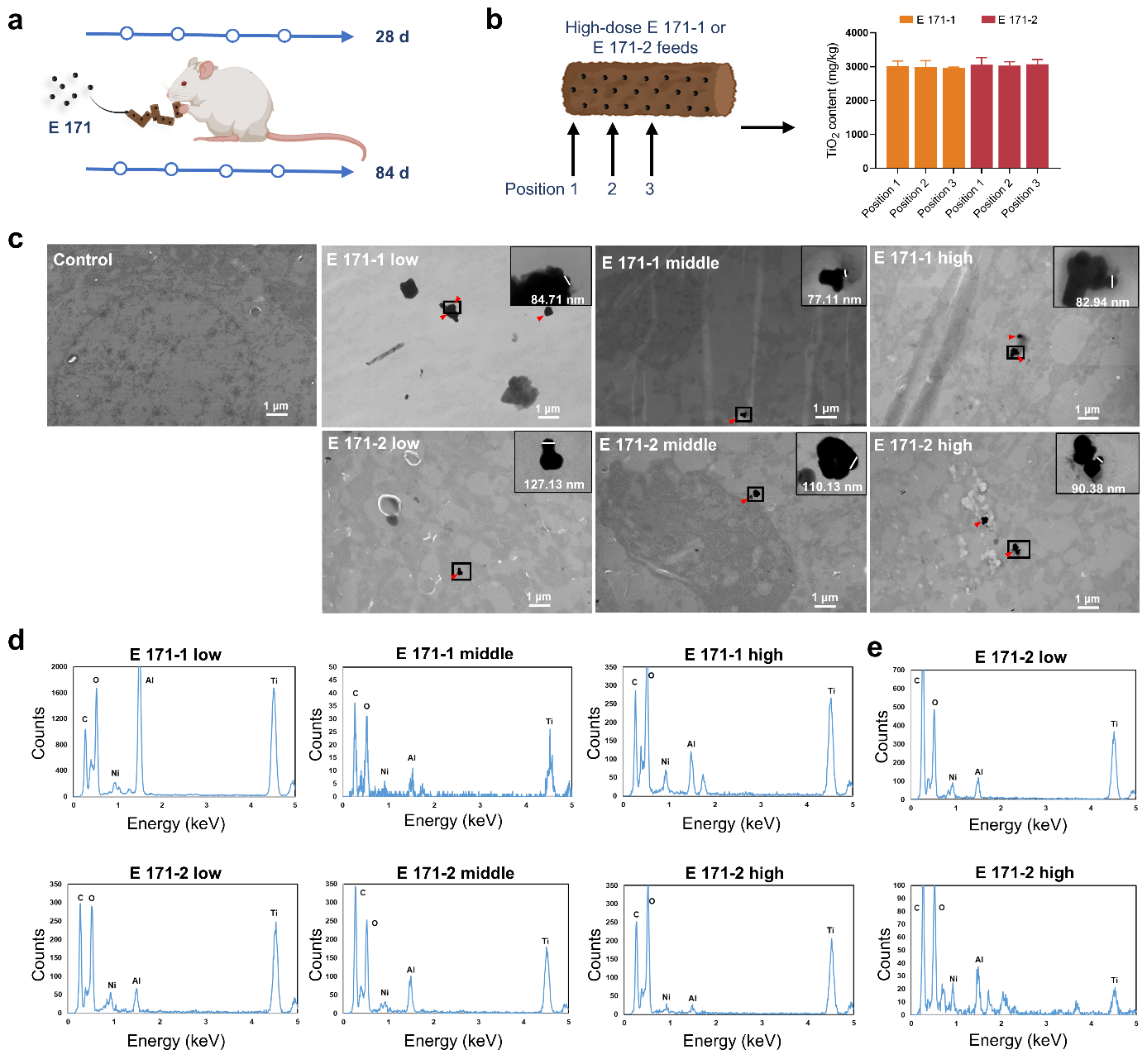


**Figure S1.** Experimental design and brain tissue distribution of E 171 following oral exposure. (a) Schematic representation of oral exposure to E 171 under subacute (28-day) and subchronic (84-day) conditions in mice. (b) ICP-MS analysis of randomly selected feed samples from the high-dose E 171 groups, showing consistent TiO_2_ content across different sampling positions. (c) Representative scanning electron microscopy images of cortical regions in mice following 84-d exposure to E 171-1 and E 171-2. (d) Energy dispersive X-ray spectroscopy analysis of the cortex in the brain of mice after 84 days of E 171-1 and E 171-2 exposure. (e) Energy dispersive X-ray spectroscopy analysis of the hippocampus in the brain of mice after 84 days of E 171-2 exposure. The presence of elemental nickel and aluminum is due to the nickel grid and the aluminum worktop, respectively.


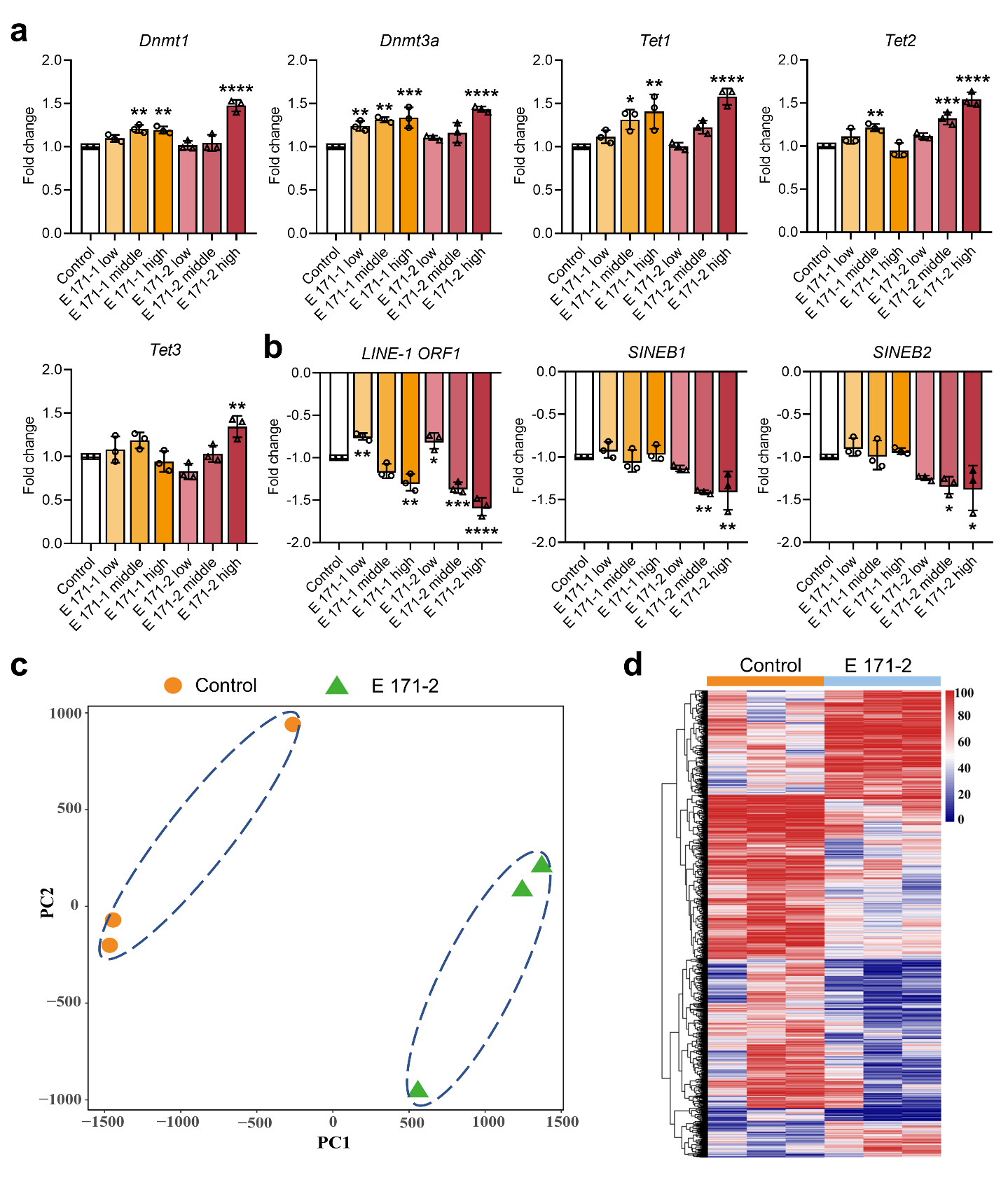


**Figure S2.** Subchronic exposure to E 171 causes epigenetic changes. (a) Gene expression of the *Dnmt* (*Dnmt1* and *Dnmt3a*) and *Tet* (*Tet1*, *Tet2*, and *Tet3*) families in the brain of mice exposed to E 171-1 and E 171-2 for 84 d. *n* = 8; *n* indicates the number of mice in each group. Statistical analysis was performed through one-way ANOVA followed by Dunnett’s test. (b) Gene expression of *LINE-1 ORF1*, *SINEB1*, and *SINEB2* in the brain of mice exposed to E 171-1 and E 171-2 for 84 d. *n* = 8; *n* indicates the number of mice in each group. Statistical analysis was performed through one-way ANOVA followed by Dunnett’s test. (c) Principal component analysis plots of CpG sites in control and middle-dose E 171-2 groups. (d) Clustering heat map of differentially methylated sites in the mCG context in control and middle-dose E 171-2 groups. *n* indicates the number of mice. Compared with the control group, ^*^*P* < 0.05, ^**^*P* < 0.01, ^***^*P* < 0.001, ^****^*P* < 0.0001.


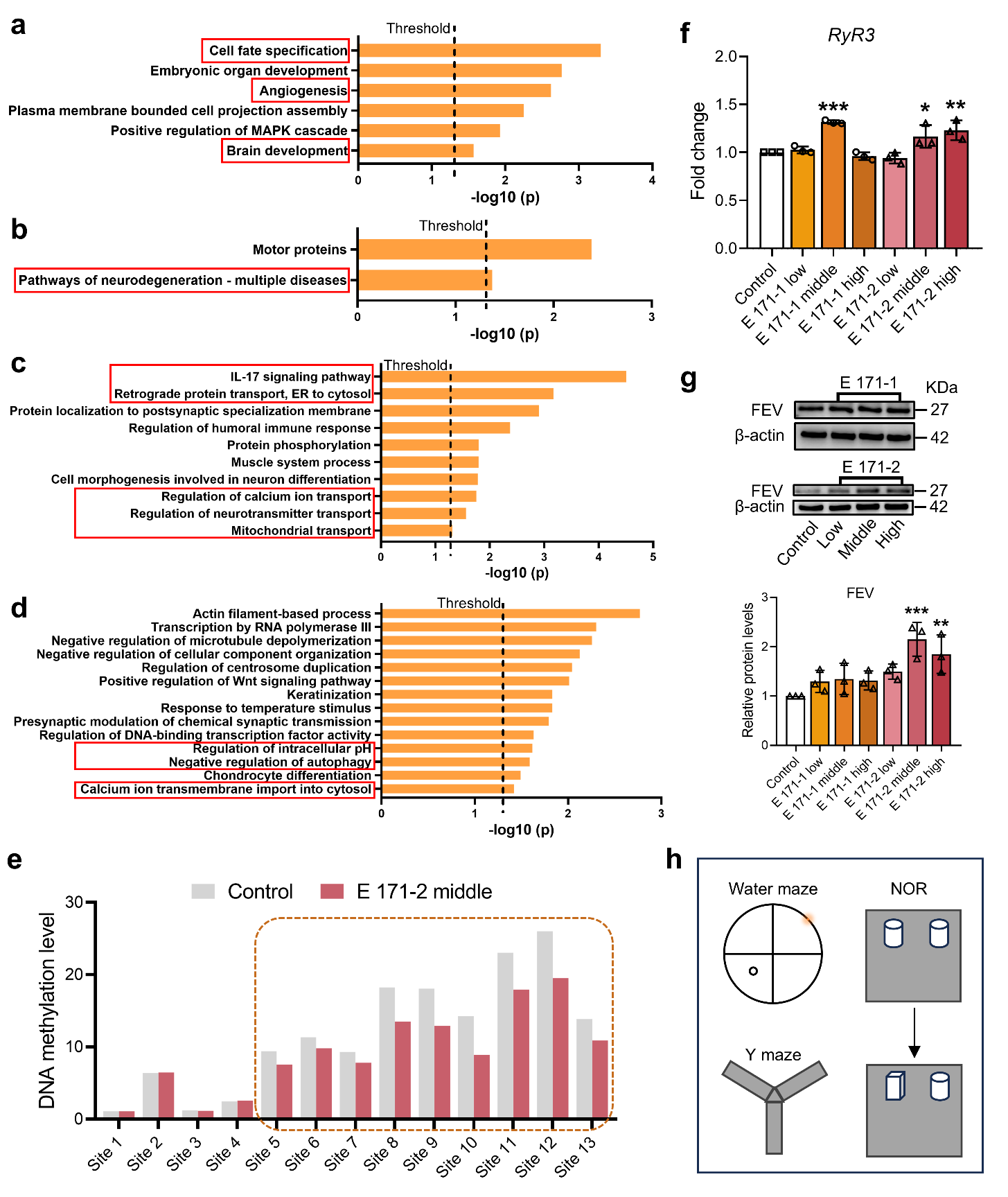


**Figure S3.** Subchronic exposure to E 171 causes epigenetic changes and the risk of neurodegenerative diseases. (a) Pathway enrichment analysis of DMGs in promoter regions within mCG contexts based on GO database. (b) Pathway enrichment analysis of DMGs in promoter regions within mCG contexts based on the KEGG database. (c) Pathway enrichment analysis of DMGs in promoter regions within mCHG contexts based on GO and KEGG databases. (d) Pathway enrichment analysis of DMGs in promoter regions within mCHH contexts based on GO and KEGG databases. (e) Changes in DNA methylation levels in the *RyR3* promoter region in the brain of mice exposed to E 171 for 84 d. (f) Gene expression of the *RyR3* in the brain of mice exposed to E 171-1 and E 171-2 for 84 d. Statistical analysis was performed through one-way ANOVA followed by Dunnett’s test. (g) Changes in protein expression of FEV in the brain of mice exposed to E 171-1 and E 171-2 for 84 d. Statistical analysis was performed through one-way ANOVA followed by Dunnett’s test. (h) Schematic diagram for performing behavioral tests on mice. Compared with the control group, ^*^*P* < 0.05, ^**^*P* < 0.01, ^***^*P* < 0.001.


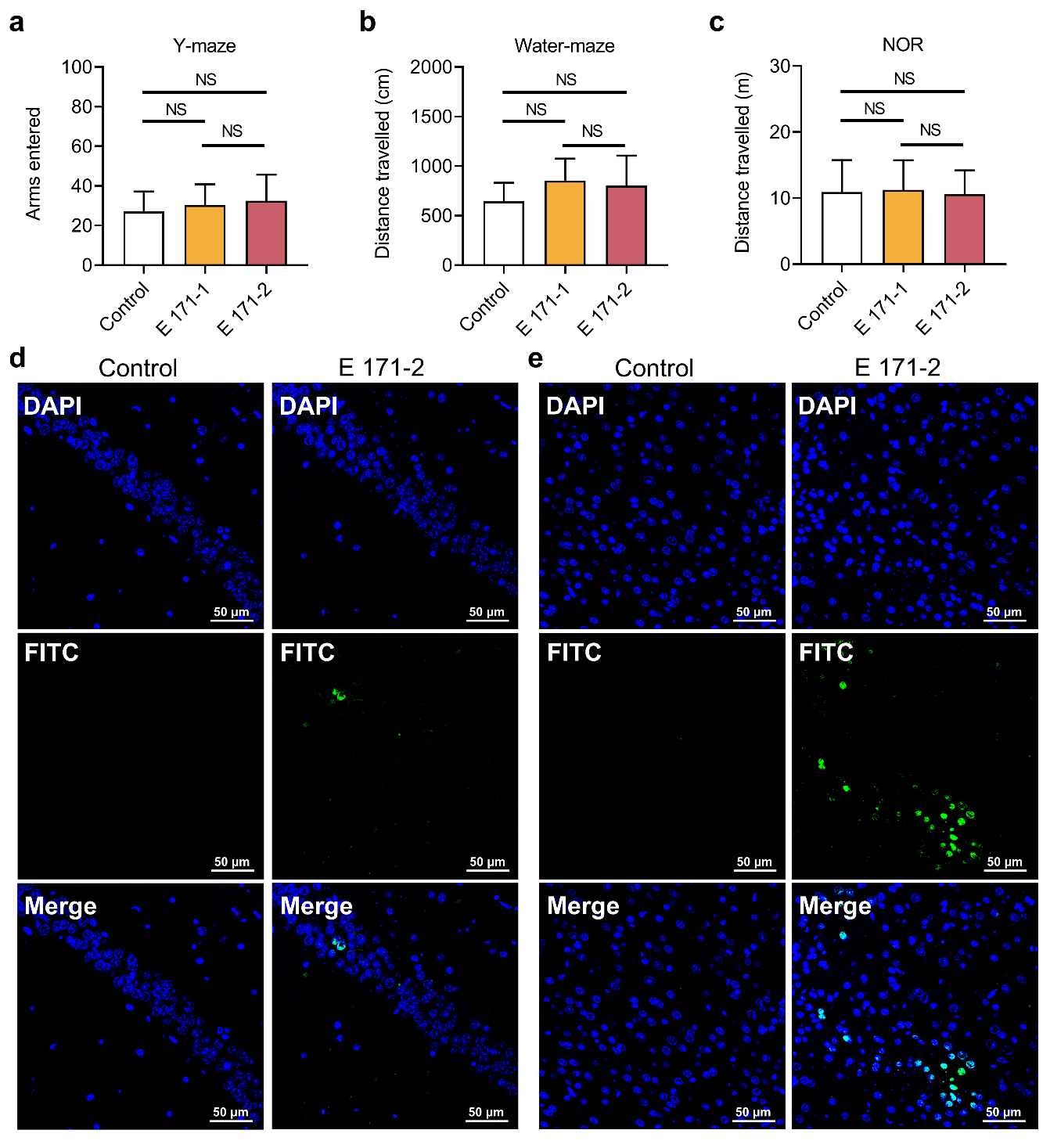


**Figure S4.** Subchronic exposure to E 171 causes apoptosis in mouse brain. (a) The total number of arm entries in the Y-maze. *n* = 8; *n* indicates the number of mice in each group. Statistical analysis was performed through one-way ANOVA with Tukey’s multiple comparison tests. (b) The total distance traveled in the water maze. *n* = 8; *n* indicates the number of mice in each group. Statistical analysis was performed through one-way ANOVA with Tukey’s multiple comparison tests. (c) The total distance traveled in the NOR area. *n* = 8; *n* indicates the number of mice in each group. Statistical analysis was performed through one-way ANOVA with Tukey’s multiple comparison tests. (d) Representative images of TUNEL staining in the CA1 region of the hippocampus from mice exposed to middle-dose E 171-2 for 84 d. (e) Representative images of TUNEL staining in the brain’s cerebral cortex region of mice exposed to middle-dose E 171-2 for 84 d.


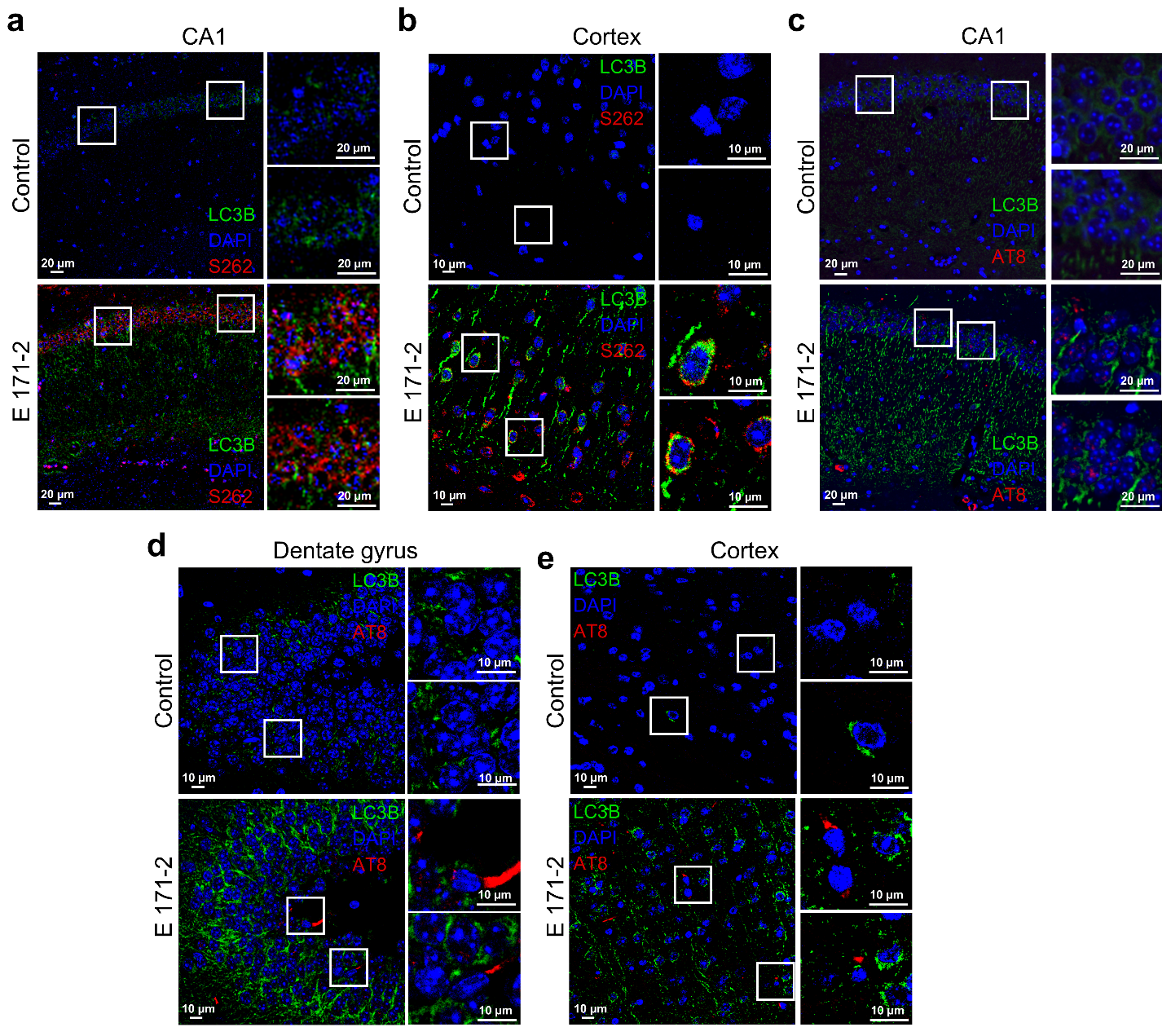


**Figure S5.** Subchronic exposure to E 171 causes autophagy defects and AD-related pathogenic protein accumulation. (a) Representative immunofluorescence images of LC3B and pTau(S262) in the hippocampal CA1 region of mice exposed to middle-dose E 171-2 for 84 d. (b) Representative immunofluorescence images of LC3B and pTau(S262) in the brain’s cerebral cortex region of mice exposed to middle-dose E 171-2 for 84 d. (c) Representative immunofluorescence images of LC3B and pTau(AT8) in the hippocampal CA1 region of mice exposed to middle-dose E 171-2 for 84 d. (d) Representative immunofluorescence images of LC3B and pTau(AT8) in the hippocampal dentate gyrus region of mice exposed to middle-dose E 171-2 for 84 d. (e) Representative immunofluorescence images of LC3B and pTau(AT8) in the brain’s cerebral cortex region of mice exposed to middle-dose E 171-2 for 84 d.


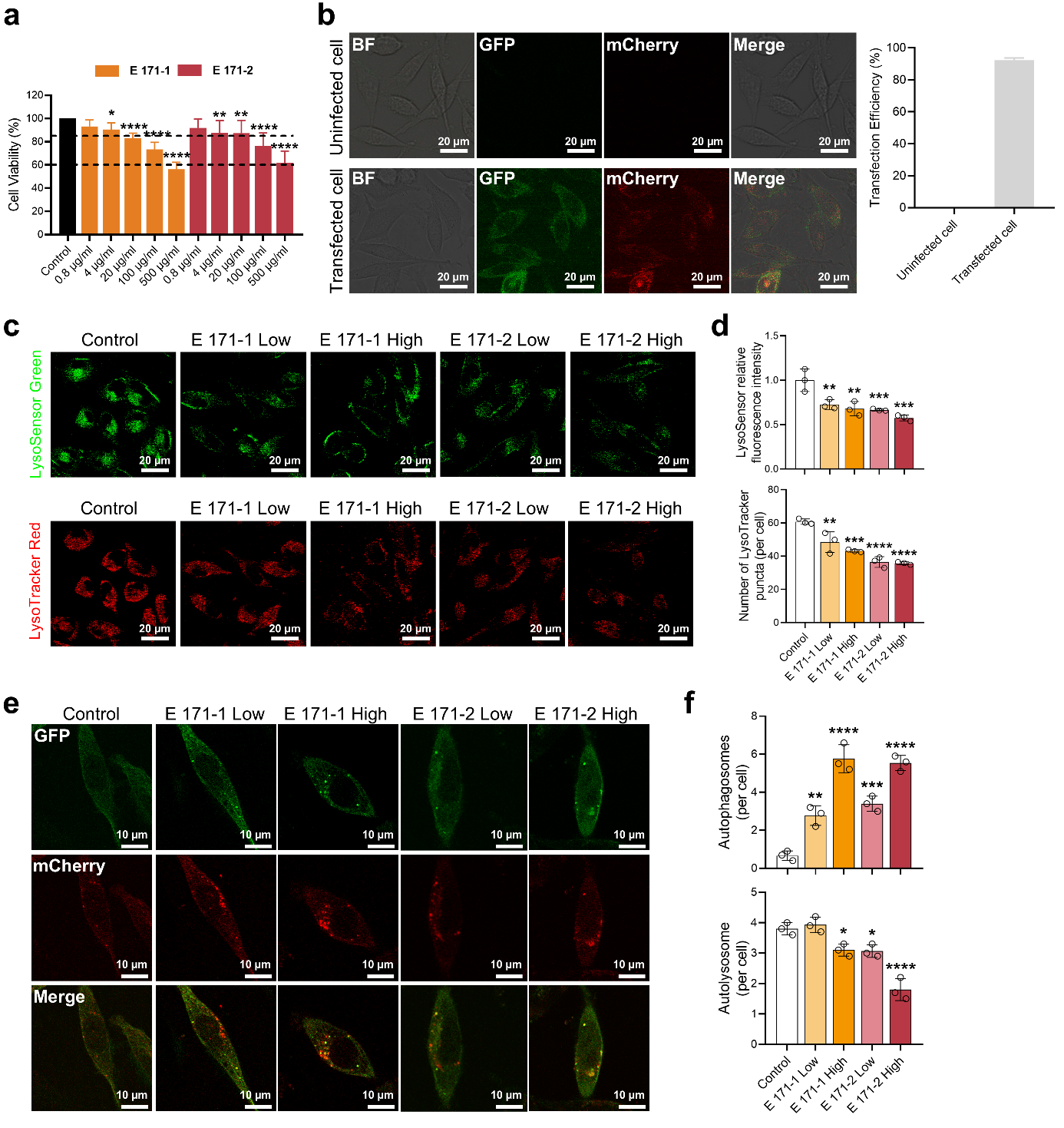


**Figure S6.** E 171 induces insufficient lysosomal acidification and impairs autophagic flux in HT22 cells. (a) The effect of E 171 exposure on the viability of HT22 cells. Statistical analysis was performed through one-way ANOVA followed by Dunnett’s test. (b) Representative images of uninfected HT22 cells and those transfected with Ad-mCherry-GFP, along with transfection efficiency. *n* = 3; *n* represents three independent experiments. In each experiment, cells from three randomly selected fields of view were analyzed. (c) Representative images of LysoSensor Green and LysoTracker Red staining in HT22 cells following exposure to E 171. (d) Relative fluorescence intensity of LysoSensor Green and the number of LysoTracker Red puncta in HT22 cells after exposure to E 171. *n* = 3; *n* represents three independent experiments. In each experiment, cells from three randomly selected fields of view were analyzed. Statistical analysis was performed through one-way ANOVA followed by Dunnett’s test. (e) Representative images of Ad-mCherry-GFP transfected HT22 cells exposed to E 171. In the merged picture, yellow fluorescent puncta represent autophagosomes and red puncta represent autolysosomes. (f) The number of autophagosomes and autolysosomes per cell exposed to E 171. *n* = 3; *n* represents three independent experiments. In each experiment, cells from three randomly selected fields of view were analyzed. Statistical analysis was performed through one-way ANOVA followed by Dunnett’s test, ^*^*P* < 0.05, ^**^*P* < 0.01, ^***^*P* < 0.001, and ^****^*P* < 0.0001.


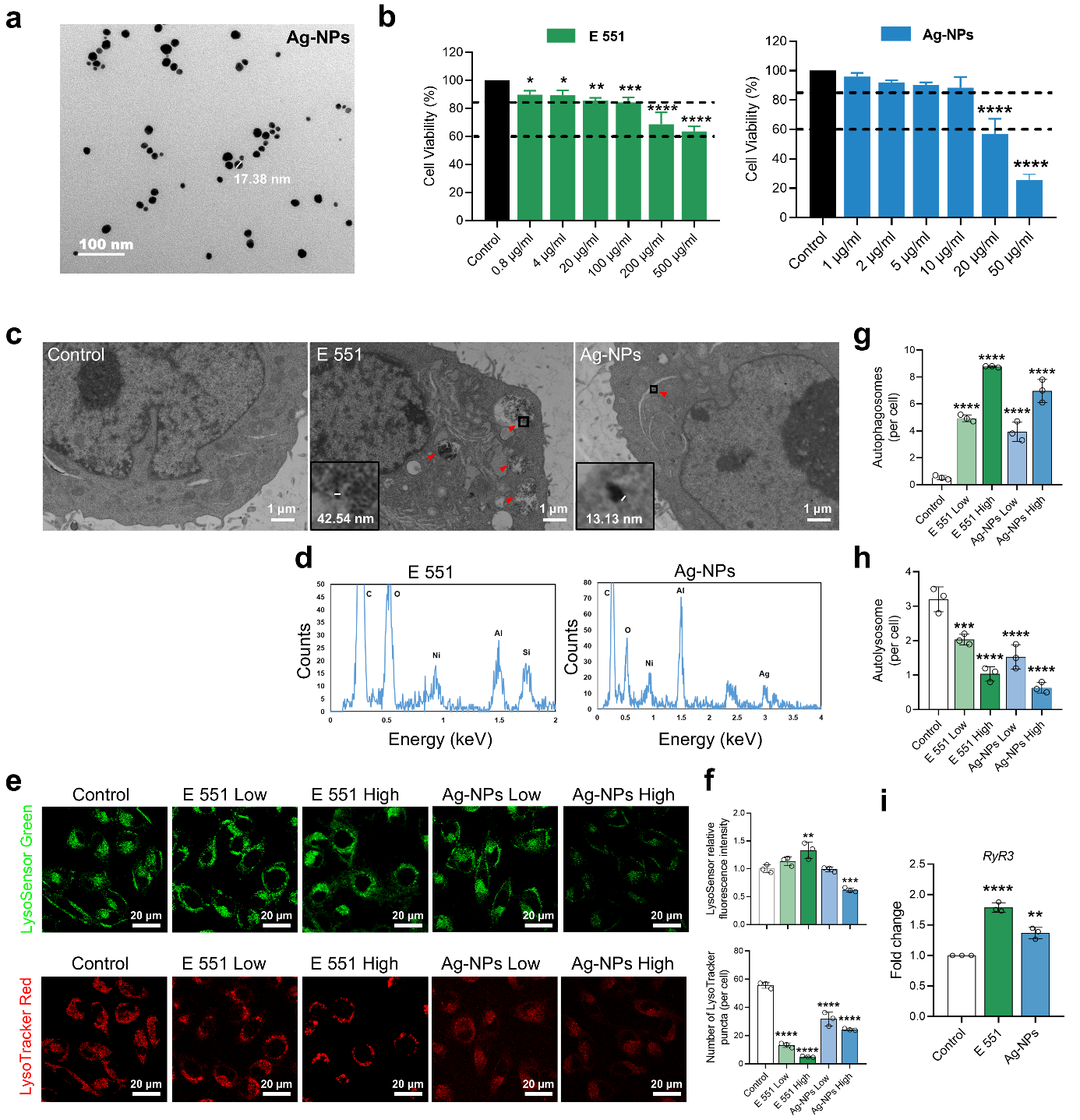


**Figure S7.** Food-grade nanoparticles trigger insufficient lysosomal acidification and impair autophagic flux in HT22 cells. (a) Representative scanning electron microscopy images of Ag-NPs. (b) The effect of E 551 or Ag-NPs exposure on the viability of HT22 cells. Statistical analysis was performed through one-way ANOVA followed by Dunnett’s test. (c) Representative scanning electron microscopy images of HT22 cells following exposure to E 551 or Ag-NPs. (d) Energy dispersive X-ray spectroscopy analysis of HT22 cells following exposure to E 551 or Ag-NPs. The presence of elemental nickel and aluminum is due to the nickel grid and the aluminum worktop, respectively. (e) Representative images of LysoSensor Green and LysoTracker Red staining in HT22 cells following exposure to E 551 or Ag-NPs. (f) Relative fluorescence intensity of LysoSensor Green and the number of LysoTracker Red puncta in HT22 cells after exposure to E 551 or Ag-NPs. *n* = 3; *n* represents three independent experiments. In each experiment, cells from three randomly selected fields of view were analyzed. Statistical analysis was performed through one-way ANOVA followed by Dunnett’s test. (g) The number of autophagosomes per cell exposed to E 551 or Ag-NPs. *n* = 3; *n* represents three independent experiments. In each experiment, cells from three randomly selected fields of view were analyzed. Statistical analysis was performed through one-way ANOVA followed by Dunnett’s test. (h) The number of autolysosomes per cell exposed to E 551 or Ag-NPs. *n* = 3; *n* represents three independent experiments. In each experiment, cells from three randomly selected fields of view were analyzed. Statistical analysis was performed through one-way ANOVA followed by Dunnett’s test. (i) Expression levels of *RyR3* after 84 days of E 551 or Ag-NPs exposure. *n* = 4; *n* indicates the number of mice in each group. Statistical analysis was performed through one-way ANOVA followed by Dunnett’s test. Compared with the control group, ^*^*P* < 0.05, ^**^*P* < 0.01, ^***^*P* < 0.001, and ^****^*P* < 0.0001.

**Table S1. Number of clean reads generated by WGBS for each sample.**

| Sample | Clean Reads | Q20 (%) | Unique mapped | Mapping ratio (%) | Conversion ratio (%) | |
| --- | --- | --- | --- | --- | --- | --- |
| Control 1 | 795616490 | 94.87 | 481268196 | 69.20 | | 99.52 |
| Control 2 | 679589812 | 94.43 | 380523088 | 63.99 | | 99.53 |
| Control 3 | 670689784 | 94.09 | 385451962 | 65.78 | | 99.53 |
| E 171-2 1 | 673821352 | 93.60 | 411895392 | 70.65 | | 99.41 |
| E 171-2 2 | 764891682 | 94.68 | 457497806 | 68.44 | | 99.40 |
| E 171-2 3 | 675056688 | 94.27 | 390159234 | 66.47 | | 99.39 |

**Table S2. Characterization of the food-grade nanoparticles in complete** **culture medium**^a)^**.**

| Food-grade nanoparticles | *d*_H_ [nm]^b)^ | Polydispersity index | ζ Potential [mV] |
| --- | --- | --- | --- |
| E 171-1 | 260.27 ± 2.61 | 0.254 ± 0.020 | −10.63 ± 0.57 |
| E 171-2 | 307.50 ± 9.88 | 0.167 ± 0.016 | −9.76 ± 0.25 |
| E 551 | 575.50 ± 25.22 | 0.280 ± 0.010 | −9.24 ± 0.75 |
| Ag-NPs | 76.83 ± 7.58 | 0.209 ± 0.004 | −8.29 ± 0.49 |

^a)^ (High-glucose Dulbecco’s modified Eagle’s medium supplemented with 100 U/mL penicillin, 100 µg/mL streptomycin, and 10% fetal bovine serum); ^b)^ (Hydrodynamic diameter, indicated by intensity).

**Table S3. The primer sequences were used for real-time quantitative PCR amplification.**

| Gene name | Forward primer | Reserve primer |
| --- | --- | --- |
| *Dnmt1* | 5'-AGAGACCAGGATAAGAAACGCA-3' | 5'-CTCCTTTGATTTCCGCCTCAAT-3' |
| *Dnmt3a* | 5'-GGCCGAATTGTGTCTTGGTG-3' | 5'-CCATCTCCGAACCACATGAC-3' |
| *Tet1* | 5'-GAGCCTGTTCCTCGATGTGG -3' | 5'-CAAACCCACCTGAGGCTGTT-3' |
| *Tet2* | 5'-CTCCTCTCCAAGCCGTGACAATG-3' | 5'-GGCGTGGTTCTCCGATTCTGC-3' |
| *Tet3* | 5'-TGCGGTGCCTCCTTCTCCTTC-3' | 5'-CCTCCTCCTCCTTCGGATTGTCTC-3' |
| *LINE-1 ORF1* | 5'-GAACCAAGACCACTCACCATCA-3' | 5'-CCCTGGACTGGGCGAAGT-3' |
| *SINEB1* | 5'-GTGGCGCACGCCTTTAATC-3' | 5'-GACAGGGTTTCTCTGTGTAG-3' |
| *SINEB2* | 5'-GAGATGGCTCAGTGGTTAAG-3' | 5'-CTGTCTTCAGACACTCCAG-3' |
| *RyR3* | 5'-AGTTTGTCAGTGGAAACAATCG-3' | 5'-TTTGTCTTCATGTTGCATCTCG-3' |
| *Rps29* | 5'-GGAGTCACCCACGGAAGT-3' | 5'-TCCATTCAAGGTCGCTTAGTC-3' |
| *Gapdh* | 5'-AGGTCGGTGTGAACGGATTTG-3' | 5'-TGTAGACCATGTAGTTGAGGTCA-3' |
